# Serum or Plasma (and Which Plasma), That Is the Question

**DOI:** 10.1021/acs.jproteome.1c00935

**Published:** 2022-03-10

**Authors:** Alessia Vignoli, Leonardo Tenori, Cristina Morsiani, Paola Turano, Miriam Capri, Claudio Luchinat

**Affiliations:** † Magnetic Resonance Center (CERM), University of Florence, 50019 Sesto Fiorentino, Italy; ‡ Department of Chemistry “Ugo Schiff”, University of Florence, 50019 Sesto Fiorentino, Italy; § DIMES − Department of Experimental, Diagnostic and Specialty Medicine, University of Bologna, 40126 Bologna, Italy; ∥ Consorzio Interuniversitario Risonanze Magnetiche Metallo Proteine (CIRMMP), 50019 Sesto Fiorentino, Italy

**Keywords:** collection tubes, citrate, EDTA, serum, plasma, NMR, metabolomics, lipidomics

## Abstract

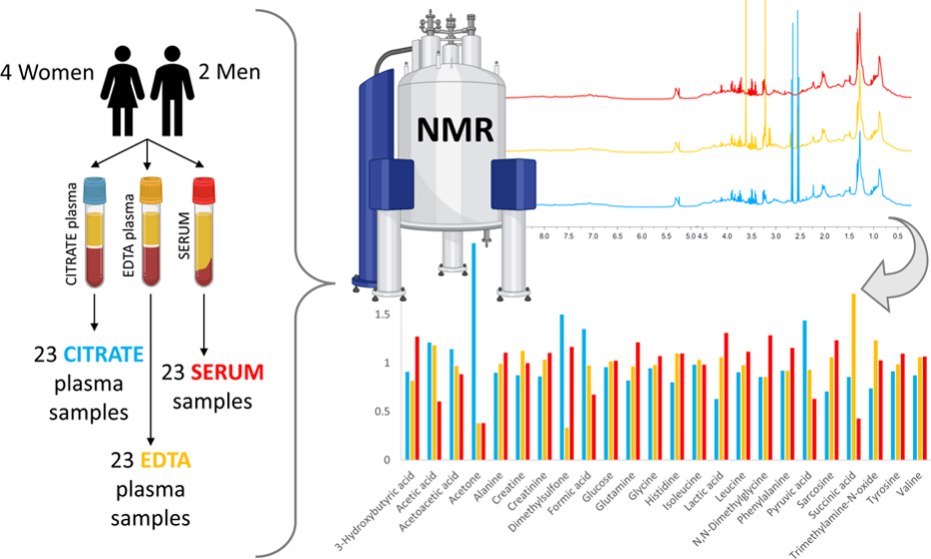

Blood derivatives are the biofluids of choice for metabolomic clinical studies since blood can be collected with low invasiveness and is rich in biological information. However, the choice of the blood collection tubes has an undeniable impact on the plasma and serum metabolic content. Here, we compared the metabolomic and lipoprotein profiles of blood samples collected at the same time and place from six healthy volunteers but using different collection tubes (each enrolled volunteer provided multiple blood samples at a distance of a few weeks/months): citrate plasma, EDTA plasma, and serum tubes. All samples were analyzed via nuclear magnetic resonance spectroscopy. Several metabolites showed statistically significant alterations among the three blood matrices, and also metabolites’ correlations were shown to be affected. The effects of blood collection tubes on the lipoproteins’ profiles are relevant too, but less marked. Overcoming the issue associated with different blood collection tubes is pivotal to scale metabolomics and lipoprotein analysis at the level of epidemiological studies based on samples from multicenter cohorts. We propose a statistical solution, based on regression, that is shown to be efficient in reducing the alterations induced by the different collection tubes for both the metabolomic and lipoprotein profiles.

## 1 Introduction

Blood, under the form of plasma and serum, is the biofluid of choice for clinical studies in general, particularly as regards metabolomics and lipidomics.^1^ Blood can be collected with low invasiveness and is rich in biological information. Blood derivatives contain metabolites as well as lipoproteins secreted by different tissues in response to various physiological stimuli, conditions, or stressors.^2^ As a consequence, serum and plasma are sensitive to health or diseased conditions, genetic variations, environmental factors, lifestyle, nutrition habits, and drugs, and they can provide important information at a systemic level. For all of these reasons, blood derivatives are often collected and biobanked for future scientific studies. However, serum or plasma samples represent a real and important resource only if collected and stored with appropriate procedures since the preanalytical phase has an impact on the human metabolome/lipoproteome stability and composition.^3^

The choice of the blood collection tubes is a key step in the preanalytical phase and influences all of the following stages. Serum is obtained after clotting by centrifugation, which allows the removal of fibrin clots, blood cells, and related coagulation factors, whereas plasma samples are obtained by adding anticoagulants (i.e., EDTA, citrate, heparin) before removal of blood cells by centrifugation.

Understanding whether the use of different blood derivatives affects the metabolome and the lipoproteins’ profile is critical to successfully translate metabolomic and lipoproteomic studies at an epidemiological level. Several studies have already investigated differences in the metabolomic profiles associated with the use of various blood collection tubes, but most of them focus on differences between EDTA plasma, heparin plasma, and serum.^1,3−13^ Bernini et al. evaluated serum, EDTA, and citrate plasma in terms of metabolome stability under different preanalytical conditions to define appropriate standard operating procedures for metabolomics and biobanks.^14^ Barton et al. conducted a study to determine how the addition of citrate or EDTA affects a metabolomic classification analysis.^15^ Recently, Sotelo-Orozco and coauthors published a study investigating how blood collected as serum differs from samples collected as ACD plasma, citrate plasma, EDTA plasma, fluoride plasma, or heparin plasma.^16^ Conversely, the effects of different blood collection tubes on the lipidome and/or lipoproteome are quite unexplored. Only recently, Wolrab et al. described, via mass spectrometry measurements, the influence of blood collection tubes (heparin plasma, EDTA plasma, and serum), sample collection site, and sample origin from a lipidomic point of view.^17^

**Figure 1:**
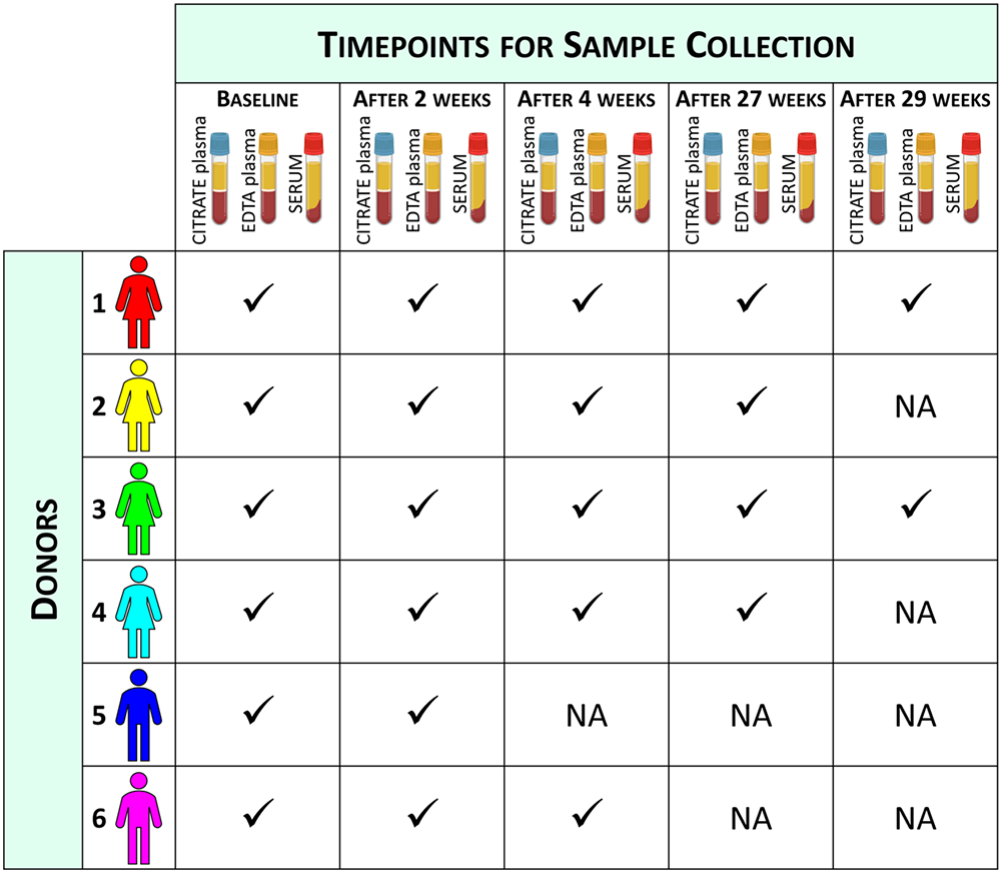
Scheme of the study design.

In the present study, we sought to deeply characterize how the NMR-based metabolomic and lipoproteomic profiles are affected by the use of three different blood collection tubes: citrate plasma tubes, EDTA plasma tubes, and serum tubes (Figure 1). Moreover, we propose a statistical approach that can overcome the differences present among the different blood derivatives.

## 2 Materials and Methods

### 2.1 Study Cohort and Sample Collection

Blood samples were collected from six healthy donors: four women of age 36, 31, 28, and 26 years, respectively, and two men of age 28 and 35 years, respectively. Each donor was subjected to multiple blood samplings over a time frame of 6 months (Figure 1): baseline, after 2 weeks, after 4 weeks, after 27 weeks, and after 29 weeks. Not all donors provided all of the five blood samples foreseen by the study protocol; thus, the total number of samples per group is 23. The Ethical Committee of S. Orsola Hospital (Bologna, Italy) approved the study protocol (EM/26/2014/U).

Serum, EDTA plasma, and citrate plasma collection tubes were provided by Greiner Bio-One (Kremsmünster, Austria). Serum samples were collected from a peripheral vein in 8 mL sterile vacutainers containing gel separator and clot activator. Each sample was allowed to clot in an upright position for 30–60 min at room temperature and then was centrifuged at 794*g* for 20 min at 4 °C. Serum was recovered, transferred into prelabeled cryovials, and stored at −80 °C within 2 h from blood collection.

EDTA plasma samples were collected from a peripheral vein in 9 mL sterile vacutainers containing tri-potassium ethylenediaminetetraacetate (K_3_EDTA). Citrate plasma samples were collected in 3.5 mL vacutainers containing a buffered trisodium citrate solution (3.2%). Each plasma sample was centrifuged at 2000*g* for 20 min at 4 °C and then plasma was recovered, transferred into prelabeled cryovials, and stored at −80 °C within 2 h from blood collection.

### 2.2 NMR Analysis

Blood plasma and serum samples were prepared following standard protocols.^18^ All NMR spectra were acquired using a Bruker 600 MHz spectrometer (Bruker BioSpin) operating at 600.13 MHz proton Larmor frequency supplied with an automatic and refrigerated (6 °C) sample changer (SampleJet, Bruker BioSpin), and a BTO 2000 thermocouple utilized for temperature stabilization (∼0.1 K at the sample). Before starting the NMR acquisition, each plasma/serum sample was maintained inside the NMR probe head for at least 300 s to reach and equilibrate at the temperature of 310 K. To ensure high spectral quality and reproducibility, the spectrometer was calibrated daily following strict standard operating procedures that include quality control of the absolute temperature, the solvent suppression, and the quantification of a reference sample.

A standard nuclear Overhauser effect spectroscopy pulse sequence NOESY 1Dpresat was applied to detect signals of low- and high-molecular-weight molecules present in each sample in concentrations above the NMR detection limit.

A detailed description of sample preparation procedures, instrument configuration, and NMR parameters setting can be retrieved from our previous publication.^19^

### 2.3 Spectral Processing and Metabolites/Lipoproteins Quantification

A line-broadening factor of 0.3 Hz was applied to each free induction decay before Fourier transform. Transformed spectra were corrected for phase and baseline distortions with automatic routine and calibrated (anomeric glucose signal δ 5.24 ppm) using TopSpin 3.6 (Bruker BioSpin GmbH, Rheinstetten, Germany).

A panel of 34 metabolites were unambiguously identified and quantified using a Bruker IVDr Quantification in Plasma/Serum B.I.Quant-PS platform (version 2.0.0). The quantification is obtained via the fitting of pre-set ^1^H NOESY signals of metabolites. The regions of glycoproteins that comprise both the NMR signals for GlycA at δ 2.04 and GlycB at δ 2.08 were quantified via peak integration.

**Figure 2:**
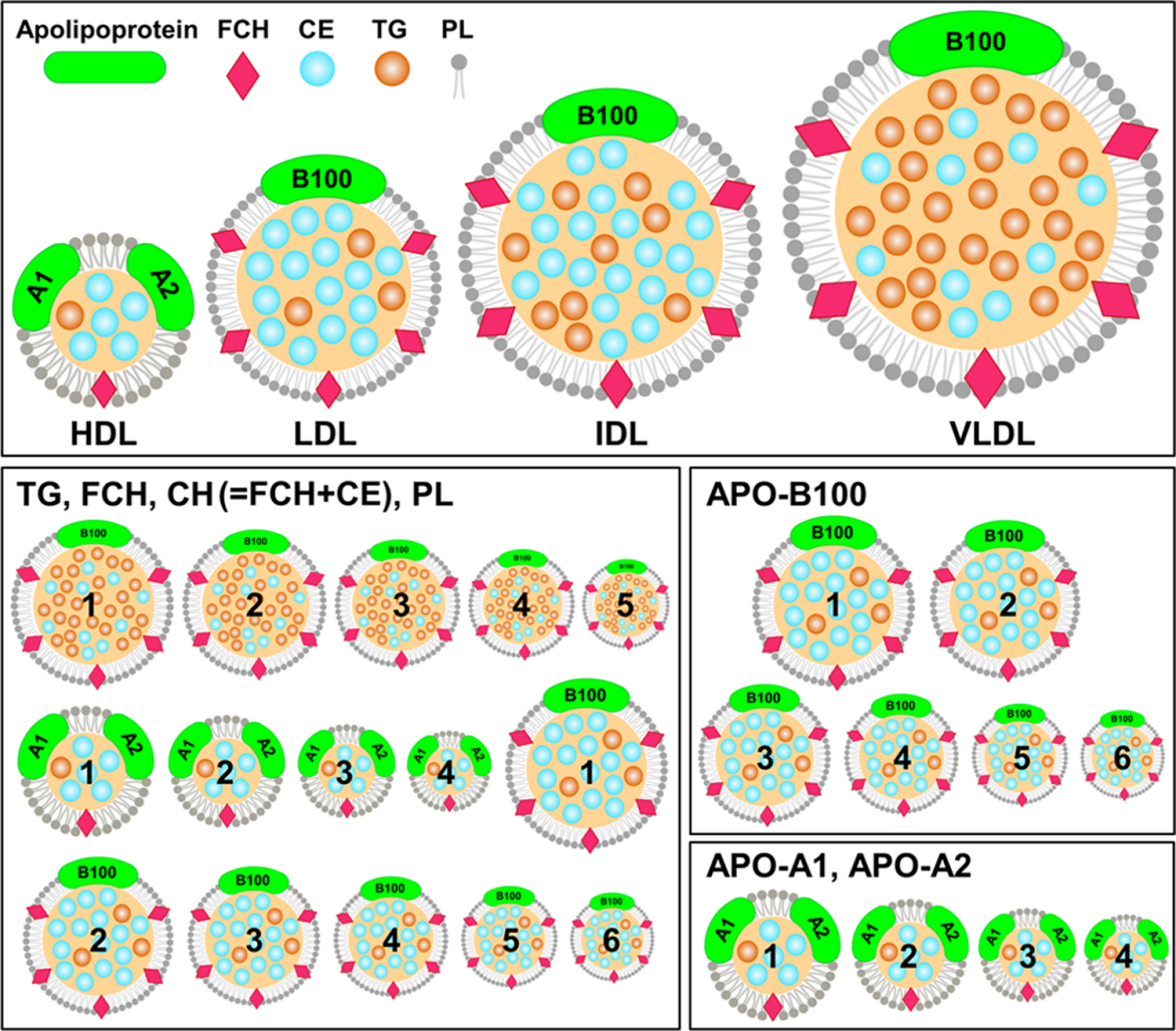
Schematic representation of the lipoprotein-related parameters measured using the Bruker IVDr Lipoprotein Subclass Analysis platform. The top panel represents the quantified lipoprotein main fractions, the bottom three panels report the parameters quantified for each lipoprotein subfraction. FCH: free cholesterol (cholesterol presents in the phospholipid membrane layer), CE: cholesterol esters, CH: sum of FCH and CE, TG: triglycerides, PL: phospholipids.

Identification and quantification of 112 lipoprotein-related parameters (Figure 2) were performed utilizing the Bruker IVDr Lipoprotein Subclass Analysis platform (version 1.0.0). This approach utilizes a PLS regression model to perform lipoprotein subclass analysis on ^1^H NMR NOESY spectra.^20,21^ Using this platform, information could be extracted about the content of triglycerides, cholesterol, free cholesterol, phospholipids, Apo-A1, Apo-A2, and Apo-B100 of the main VLDL, IDL, LDL, and HDL classes, six VLDL subclasses (VLDL-1 to VLDL-6 sorted according to increasing density and decreasing size, respectively), six LDL subclasses (LDL-1 to LDL-6), and four HDL-subclasses (HDL-1 to HDL-4).

### 2.4 Statistical Analysis

All data analyses were performed in the “R” statistical environment^22^ (Microsoft R Open, version 4.0.2). Regarding metabolites, values lower than the limit of quantification (LOQ) were imputed by means of a Random Forest approach as implemented in the R package “missForest”^23^ using 500 trees and default parameters, covariates with more than 25% of observation under the LOQ were excluded from the present analysis; thus, nine metabolites were removed (Table S1). Moreover, since citrate is obviously added in the vacutainers suitable for the collection of citrate plasma also this metabolite was removed from the dataset. The concentrations of metabolites and lipoprotein-related parameters quantified in citrate plasma samples were corrected for a dilution factor of 3.2%.

Principal component analysis (PCA) was used to obtain a multivariate overview of metabolomic/lipoproteomic data. PCA was calculated with the basic R function “prcomp” present in package “stats”, and data were auto-scaled (scaled by standard deviation and centered on the mean) prior to PCA.

Repeated measures ANOVA (R function “aov”, package “stats”) was used to infer differences between metabolites and lipoprotein-related parameters of the groups of interest. Spearman correlations were calculated between metabolomic/lipoproteomic data using the function “corr.test” of the R package “psych”. *p*-values of univariate analyses were adjusted for multiple testing using the false discovery rate (FDR) procedure with the Benjamini–Hochberg^24^ correction at α = 0.05. This univariate analysis was extended to the integrated region of glycoproteins.

To statistically remove the effects associated with blood collection tubes, the “aov” R function was used to calculate regression models. Each continuous variable (concentrations of metabolites and lipoproteins, 136 variables in total) was separately regressed against the categorical variable indicating for each sample the collection tube used (category “citrate plasma” encoded as value 1, category “EDTA plasma” encoded as value 2, category “serum” encoded as value 3). The residuals originated from each regression were reorganized into two new matrices, one for metabolites (24 variables) and one for lipoproteins (112 variables). The matrices of the residuals of both metabolites and lipoproteins were then analyzed via PCA. The cluster validity was assessed by calculating the Calinski–Harabasz (CH) index (R function “intCriteria”, package “clusterCrit”), higher value of CH index implies that the clusters are dense and well separated.

## 3 Results and Discussion

**Figure 3:**
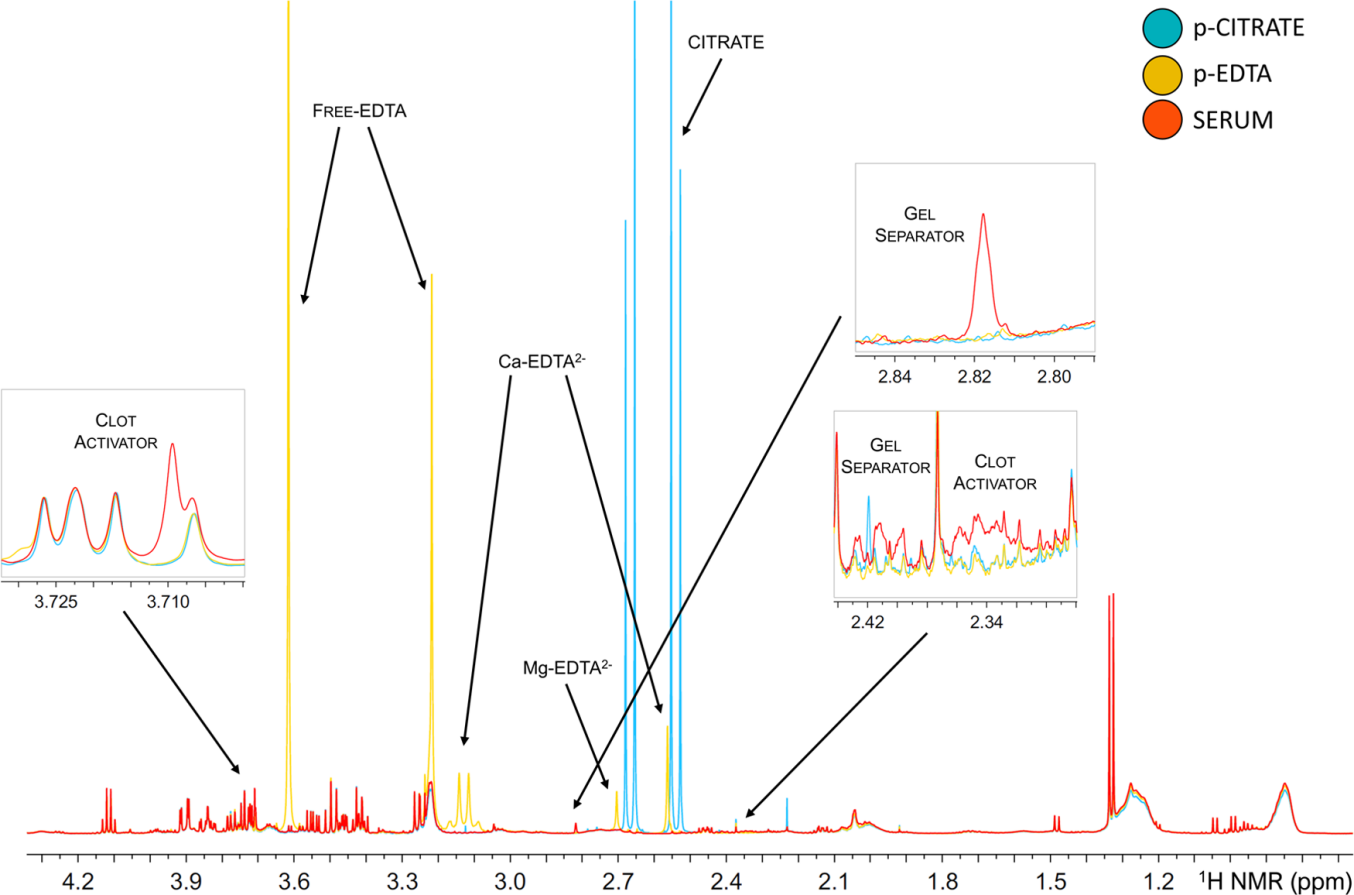
Example of the upfield region of the ^1^H NMR spectrum for each of the three blood derivatives: serum (red), EDTA plasma (yellow), and citrate plasma (blue). Additive signals for each blood collection tube are indicated.

Collection tubes have a relevant impact on the NMR-based metabolomic profiles of blood plasma and serum samples. Plasma is obtained by mixing blood with anticoagulants to inhibit clotting; however, both EDTA and citrate give rise to NMR peaks (Figure 3). EDTA plasma samples show a complex and strong set of NMR peaks in the region between 2.5 and 3.6 ppm arising from the protons of free EDTA itself, as well as the ones originating from the protons of chelated EDTA (Ca–EDTA^2–^ and Mg–EDTA^2–^ complexes). Citrate presents only a multiplet (and its satellites) at 2.6 ppm; however, its use as an additive prevents obtaining information on one relevant player in the TCA cycle. Serum is often considered the gold standard for metabolomics as it is prepared from coagulated blood and requires no additives; however, today, the most commonly used blood collection tubes for serum contains separator gels that form a barrier between packed cells and serum during centrifugation, improving analyte stability and providing an easier separation,^25^ and clot-activating agents that reduced the clotting time down to 30 min. However, both separator gels and clotting agents produce signals in ^1^H NMR spectra (Figure 3), and therefore, they have no negligible effects on the overall metabolic fingerprint. All of the above-mentioned aspects need to be carefully evaluated when designing an experiment where metabolomics analysis is planned or when samples are prepared for bank storage. In the present study, we sought to compare how the NMR-based metabolomic and lipoproteomic profiles of serum differ from the profiles of plasma in EDTA and citrate tubes, although collected under the same experimental conditions.

### 3.1 Influences of Different Blood Collection Tubes on Metabolites

A total of 24 metabolites were identified and quantified in all samples and used for our analyses. These include amino acids and their derivatives (alanine, creatine, creatinine, glutamine, glycine, histidine, isoleucine, leucine, *N*,*N*-dimethylglycine, phenylalanine, sarcosine, tyrosine, valine), carboxylic acids (acetic acid, formic acid, lactic acid, succinic acid), keto acids and derivatives (3-hydroxybutyric acid, acetoacetic acid, acetone, pyruvic acid), and other compounds such as glucose, dimethylsulfone, and trimethylamine-N-oxide. The median, standard deviation, and 95% confidence interval of all metabolites, for each tube type, are provided in Table 1.

**Table 1:** Blood Metabolites and Main Lipoprotein-Related Parameters Quantified via ^1^H NMR Spectroscopy^a^

	CITRATE plasma	EDTA plasma	SERUM	*p*-value citrate-EDTA	*p*-value citrate-serum	*p*-value EDTA-serum
**Metabolites (mmol/L)**						
3-hydroxybutyric acid						
mean (SD) 95% CI	5.70 × 10^–2^ (2.90 × 10^–2^) (4.40 × 10^–2^–7.00 × 10^–2^)	5.30 × 10^–2^ (3.20 × 10^–2^) (4.00 × 10^–2^–6.70 × 10^–2^)	6.90 × 10^–2^ (3.90 × 10^–2^) (5.20 × 10^–2^–8.60 × 10^–2^)	6.61 × 10^–1^	2.20 × 10^–1^	1.43 × 10^–1^
acetic acid						
mean (SD) 95% CI	5.40 × 10^–2^ (2.80 × 10^–2^) (4.20 × 10^–2^–6.60 × 10^–2^)	5.10 × 10^–2^ (2.70 × 10^–2^) (3.90 × 10^–2^–6.30 × 10^–2^)	2.90 × 10^–2^ (2.40 × 10^–2^) (1.90 × 10^–2^–4.00 × 10^–2^)	6.47 × 10^–1^	5.40 × 10^–4^	2.00 × 10^–3^
acetoacetic acid						
mean (SD) 95% CI	2.50 × 10^–2^ (1.50 × 10^–2^) (1.80 × 10^–2^–3.10 × 10^–2^)	2.00 × 10^–2^ (1.20 × 10^–2^) (1.50 × 10^–2^–2.50 × 10^–2^)	1.90 × 10^–2^ (1.50 × 10^–2^) (1.20 × 10^–2^–2.50 × 10^–2^)	2.46 × 10^–1^	1.59 × 10^–1^	7.84 × 10^–1^
acetone						
mean (SD) 95% CI	1.14 × 10^–1^ (4.50 × 10^–2^) (9.40 × 10^–2^–1.34 × 10^–1^)	2.00 × 10^–2^ (9.00 × 10^–3^) (1.60 × 10^–2^–2.40 × 10^–2^)	2.40 × 10^–2^ (1.10 × 10^–2^) (2.00 × 10^–2^–2.90 × 10^–2^)	8.21 × 10^–13^	1.25 × 10^–12^	1.43 × 10^–1^
alanine						
mean (SD) 95% CI	3.33 × 10^–1^ (5.90 × 10^–2^) (3.07 × 10^–1^–3.58 × 10^–1^)	3.61 × 10^–1^ (6.40 × 10^–2^) (3.33 × 10^–1^–3.89 × 10^–1^)	4.08 × 10^–1^ (6.60 × 10^–2^) (3.79 × 10^–1^–4.36 × 10^–1^)	1.02 × 10^–1^	2.06 × 10^–5^	5.72 × 10^–3^
creatine						
mean (SD) 95% CI	1.2 × 10^–2^ (9.00 × 10^–3^) (7.00 × 10^–3^–1.60 × 10^–2^)	1.20 × 10^–2^ (1.00 × 10^–2^) (8.00 × 10^–3^–1.70 × 10^–2^)	1.20 × 10^–2^ (1.00 × 10^–2^) (7.00 × 10^–3^–1.60 × 10^–2^)	5.99 × 10^–1^	8.24 × 10^–1^	8.26 × 10^–1^
creatinine						
mean (SD) 95% CI	6.90 × 10^–2^ (1.80 × 10^–2^) (6.10 × 10^–2^–7.70 × 10^–2^)	7.20 × 10^–2^ (2.20 × 10^–2^) (6.70 × 10^–2^–8.60 × 10^–2^)	8.00 × 10^–2^ (2.10 × 10^–2^) (7.10 × 10^–2^–8.90 × 10^–2^)	6.64 × 10^–2^	1.32 × 10^–3^	3.16 × 10^–1^
dimethylsulfone						
mean (SD) 95% CI	1.10 × 10^–2^ (6.00 × 10^–3^) (9.00 × 10^–3^–1.40 × 10^–2^)	4.00 × 10^–3^ (5.00 × 10^–3^) (2.00 × 10^–3^–6.00 × 10^–3^)	9.00 × 10^–3^ (7.00 × 10^–3^) (7.00 × 10^–3^–1.20 × 10^–2^)	1.05 × 10^–10^	3.64 × 10^–2^	3.08 × 10^–7^
formic acid						
mean (SD) 95% CI	3.60 × 10^–2^ (6.00 × 10^–3^) (3.40 × 10^–2^–3.90 × 10^–2^)	2.70 × 10^–2^ (6.00 × 10^–3^) (2.40 × 10^–2^–2.90 × 10^–2^)	1.80 × 10^–2^ (5.00 × 10^–3^) (1.60 × 10^–2^–2.10 × 10^–2^)	9.43 × 10^–7^	3.78 × 10^–14^	5.80 × 10^–6^
glucose						
mean (SD) 95% CI	5.17 × 10^0^ (4.50 × 10^–1^) (4.98 × 10^0^–5.37 × 10^0^)	5.32 × 10^0^ (5.36 × 10^–1^) (5.09 × 10^0^–5.55 × 10^0^)	5.24 × 10^0^ (4.36 × 10^–1^) (5.05 × 10^0^–5.43 × 10^0^)	1.02 × 10^–1^	4.85 × 10^–1^	4.40 × 10^–1^
glutamine						
mean (SD) 95% CI	6.97 × 10^–1^ (1.18 × 10^–1^) (6.46 × 10^–1^–7.48 × 10^–1^)	7.86 × 10^–1^ (1.18 × 10^–1^) (7.35 × 10^–1^–8.37 × 10^–1^)	9.70 × 10^–1^ (1.43 × 10^–1^) (9.08 × 10^–1^–1.03 × 10^0^)	1.99 × 10^–3^	2.54 × 10^–11^	4.25 × 10^–7^
glycine						
mean (SD) 95% CI	2.54 × 10^–1^ (4.50 × 10^–2^) (2.35 × 10^–1^–2.73 × 10^–1^)	2.62 × 10^–1^ (5.30 × 10^–2^) (2.39 × 10^–1^–2.85 × 10^–1^)	2.82 × 10^–1^ (4.90 × 10^–2^) (2.61 × 10^–1^–3.03 × 10^–1^)	4.03 × 10^–1^	1.16 × 10^–3^	7.60 × 10^–2^
histidine						
mean (SD) 95% CI	7.20 × 10^–2^ (1.00 × 10^–2^) (6.80 × 10^–2^–7.70 × 10^–2^)	9.20 × 10^–2^ (1.50 × 10^–2^) (8.60 × 10^–2^–9.90 × 10^–2^)	9.30 × 10^–2^ (1.30 × 10^–2^) (8.70 × 10^–2^–9.80 × 10^–2^)	2.19 × 10^–6^	1.18 × 10^–7^	8.99 × 10^–1^
isoleucine						
mean (SD) 95% CI	5.90 × 10^–2^ (1.40 × 10^–2^) (5.30 × 10^–2^–6.50 × 10^–2^)	6.20 × 10^–2^ (1.50 × 10^–2^) (5.50 × 10^–2^–6.80 × 10^–2^)	6.10 × 10^–2^ (1.20 × 10^–2^) (5.50 × 10^–2^–6.60 × 10^–2^)	4.03 × 10^–1^	5.67 × 10^–1^	7.84 × 10^–1^
lactic acid						
mean (SD) 95% CI	1.44 × 10^0^ (6.83 × 10^–1^) (1.14 × 10^0^–1.73 × 10^0^)	2.04 × 10^0^ (8.67 × 10^–1^) (1.67 × 10^0^–2.42 × 10^0^)	2.46 × 10^0^ (7.38 × 10^–1^) (2.14 × 10^0^–2.77 × 10^0^)	2.90 × 10^–3^	1.01 × 10^–6^	7.48 × 10^–2^
leucine						
mean (SD) 95% CI	1.05 × 10^–1^ (2.00 × 10^–2^) (9.60 × 10^–2^–1.14 × 10^–1^)	1.13 × 10^–1^ (2.30 × 10^–2^) (1.03 × 10^–1^–1.23 × 10^–1^)	1.24 × 10^–1^ (2.50 × 10^–2^) (1.13 × 10^–1^–1.35 × 10^–1^)	2.07 × 10^–1^	4.97 × 10^–3^	8.52 × 10^–2^
*N*,*N*-dimethylglycine						
mean (SD) 95% CI	5.00 × 10^–3^ (1.00 × 10^–3^) (4.00 × 10^–3^–5.00 × 10^–3^)	5.00 × 10^–3^ (1.00 × 10^–3^) (5.00 × 10^–3^–6.00 × 10^–3^)	7.00 × 10^–3^ (1.00 × 10^–3^) (7.00 × 10^–3^–8.00 × 10^–3^)	1.76 × 10^–1^	9.40 × 10^–8^	4.72 × 10^–6^
phenylalanine						
mean (SD) 95% CI	4.10 × 10^–2^ (7.00 × 10^–3^) (3.70 × 10^–2^–4.40 × 10^–2^)	3.90 × 10^–2^ (6.00 × 10^–3^) (3.60 × 10^–2^–4.20 × 10^–2^)	4.90 × 10^–2^ (7.00 × 10^–3^) (4.50 × 10^–2^–5.20 × 10^–2^)	3.08 × 10^–1^	5.62 × 10^–6^	1.94 × 10^–6^
pyruvic acid						
mean (SD) 95% CI	1.35 × 10^–1^ (1.80 × 10^–2^) (1.27 × 10^–1^–1.43 × 10^–1^)	8.80 × 10^–2^ (2.00 × 10^–2^) (8.00 × 10^–2^–9.70 × 10^–2^)	6.30 × 10^–2^ (2.20 × 10^–2^) (5.40 × 10^–2^–7.30 × 10^–2^)	1.05 × 10^–10^	2.36 × 10^–14^	3.91 × 10^–5^
sarcosine						
mean (SD) 95% CI	3.00 × 10^–3^ (2.00 × 10^–3^) (2.00 × 10^–3^–4.00 × 10^–3^)	5.00 × 10^–3^ (3.00 × 10^–3^) (3.00 × 10^–3^–7.00 × 10^–3^)	4.00 × 10^–3^ (2.00 × 10^–3^) (3.00 × 10^–3^–5.00 × 10^–3^)	4.86 × 10^–2^	7.64 × 10^–2^	3.36 × 10^–1^
succinic acid						
mean (SD) 95% CI	3.00 × 10^–3^ (2.00 × 10^–3^) (2.00 × 10^–3^–4.00 × 10^–3^)	4.00 × 10^–3^ (2.00 × 10^–3^) (3.00 × 10^–3^–5.00 × 10^–3^)	4.00 × 10^–3^ (5.00 × 10^–3^) (2.00 × 10^–3^–6.00 × 10^–3^)	1.02 × 10^–1^	2.61 × 10^–1^	7.86 × 10^–1^
trimethylamine-N-oxide						
mean (SD) 95% CI	2.10 × 10^–2^ (8.00 × 10^–3^) (1.80 × 10^–2^–2.50 × 10^–2^)	2.90 × 10^–2^ (7.00 × 10^–3^) (2.50 × 10^–2^–3.20 × 10^–2^)	2.60 × 10^–2^ (1.00 × 10^–2^) (2.20 × 10^–2^–3.10 × 10^–2^)	6.94 × 10^–3^	8.09 × 10^–2^	5.27 × 10^–1^
tyrosine						
mean (SD) 95% CI	5.20 × 10^–2^ (1.00 × 10^–2^) (4.70 × 10^–2^–5.60 × 10^–2^)	5.40 × 10^–2^ (1.00 × 10^–2^) (5.00 × 10^–2^–5.80 × 10^–2^)	6.10 × 10^–2^ (1.00 × 10^–2^) (5.70 × 10^–2^–6.60 × 10^–2^)	3.75 × 10^–1^	2.45 × 10^–4^	1.37 × 10^–3^
valine						
mean (SD) 95% CI	2.32 × 10^–1^ (3.70 × 10^–2^) (2.15 × 10^–1^–2.48 × 10^–1^)	2.52 × 10^–1^ (5.20 × 10^–2^) (2.30 × 10^–1^–2.74 × 10^–1^)	2.63 × 10^–1^ (5.40 × 10^–2^) (2.40 × 10^–1^–2.86 × 10^–1^)	7.00 × 10^–2^	4.97 × 10^–3^	3.36 × 10^–1^
**Lipoprotein Parameters (mg/dL)**						
triglycerides						
mean (SD) 95% CI	6.84 × 10^1^ (2.50 × 10^1^) (5.76 × 10^1^–7.93 × 10^1^)	7.02 × 10^1^ (2.68 × 10^1^) (5.85 × 10^1^–8.18 × 10^1^)	7.15 × 10^1^ (2.82 × 10^1^) (5.92 × 10^1^–8.37 × 10^1^)	7.16 × 10^–1^	5.06 × 10^–1^	9.93 × 10^–1^
cholesterol						
mean (SD) 95% CI	1.73 × 10^2^ (2.43 × 10^1^) (1.63 × 10^2^–1.84 × 10^2^)	1.87 × 10^2^ (2.61 × 10^1^) (1.76 × 10^2^–1.99 × 10^2^)	1.90 × 10^2^ (2.90 × 10^1^) (1.78 × 10^2^–2.03 × 10^2^)	2.35 × 10^–3^	3.39 × 10^–3^	9.93 × 10^–1^
cholesterol, VLDL						
mean (SD) 95% CI	1.12 × 10^1^ (5.67 × 10^0^) (8.73 × 10^0^–1.36 × 10^1^)	1.14 × 10^1^ (5.96 × 10^0^) (8.78 × 10^0^–1.39 × 10^1^)	1.08 × 10^1^ (6.58 × 10^0^) (8.00 × 10^0^–1.37 × 10^1^)	8.70 × 10^–1^	8.00 × 10^–1^	9.93 × 10^–1^
cholesterol, IDL						
mean (SD) 95% CI	4.44 × 10^0^ (3.25 × 10^0^) (3.04 × 10^0^–5.85 × 10^0^)	6.67 × 10^0^ (3.47 × 10^0^) (5.17 × 10^0^–8.17 × 10^0^)	6.76 × 10^0^ (3.79 × 10^0^) (5.12 × 10^0^–8.40 × 10^0^)	1.33 × 10^–3^	1.61 × 10^–3^	9.95 × 10^–1^
cholesterol, LDL						
mean (SD) 95% CI	8.91 × 10^1^ (2.22 × 10^1^) (7.95 × 10^1^–9.87 × 10^1^)	9.85 × 10^1^ (2.35 × 10^1^) (8.83 × 10^1^–1.09 × 10^2^)	9.88 × 10^1^ (2.47 × 10^1^) (8.81 × 10^1^–1.09 × 10^2^)	1.33 × 10^–2^	2.90 × 10^–2^	9.95 × 10^–1^
cholesterol, HDL						
mean (SD) 95% CI	5.81 × 10^1^ (9.98 × 10^0^) (5.38 × 10^1^–6.24 × 10^1^)	6.24 × 10^1^ (1.16 × 10^1^) (5.74 × 10^1^–6.74 × 10^1^)	6.57 × 10^1^ (1.31 × 10^1^) (6.01 × 10^1^–7.14 × 10^1^)	1.03 × 10^–3^	1.12 × 10^–5^	8.16 × 10^–2^
Apo-A1						
mean (SD) 95% CI	1.50 × 10^2^ (1.53 × 10^1^) (1.44 × 10^2^–1.57 × 10^2^)	1.60 × 10^2^ (1.75 × 10^1^) (1.52 × 10^2^–1.67 × 10^2^)	1.65 × 10^2^ (2.19 × 10^1^) (1.55 × 10^2^–1.74 × 10^2^)	8.89 × 10^–4^	8.20 × 10^–5^	2.50 × 10^–1^
Apo-A2						
mean (SD) 95% CI	3.17 × 10^1^ (3.45 × 10^0^) (3.02 × 10^1^–3.32 × 10^1^)	3.44 × 10^1^ (3.76 × 10^0^) (3.28 × 10^1^–3.61 × 10^1^)	3.63 × 10^1^ (5.05 × 10^0^) (3.41 × 10^1^–3.85 × 10^1^)	3.85 × 10^–4^	1.47 × 10^–5^	1.62 × 10^–1^
Apo-B100						
mean (SD) 95% CI	6.47 × 10^1^ (1.46 × 10^1^) (5.84 × 10^1^–7.10 × 10^1^)	6.84 × 10^1^ (1.54 × 10^1^) (6.17 × 10^1^–7.50 × 10^1^)	6.82 × 10^1^ (1.57 × 10^1^) (6.14 × 10^1^–7.50 × 10^1^)	5.07 × 10^–2^	1.06 × 10^–1^	9.95 × 10^–1^

a *p*-values are adjusted for multiple testing using the FDR procedure with the Benjamini–Hochberg correction at α = 0.05.

**Figure 4:**
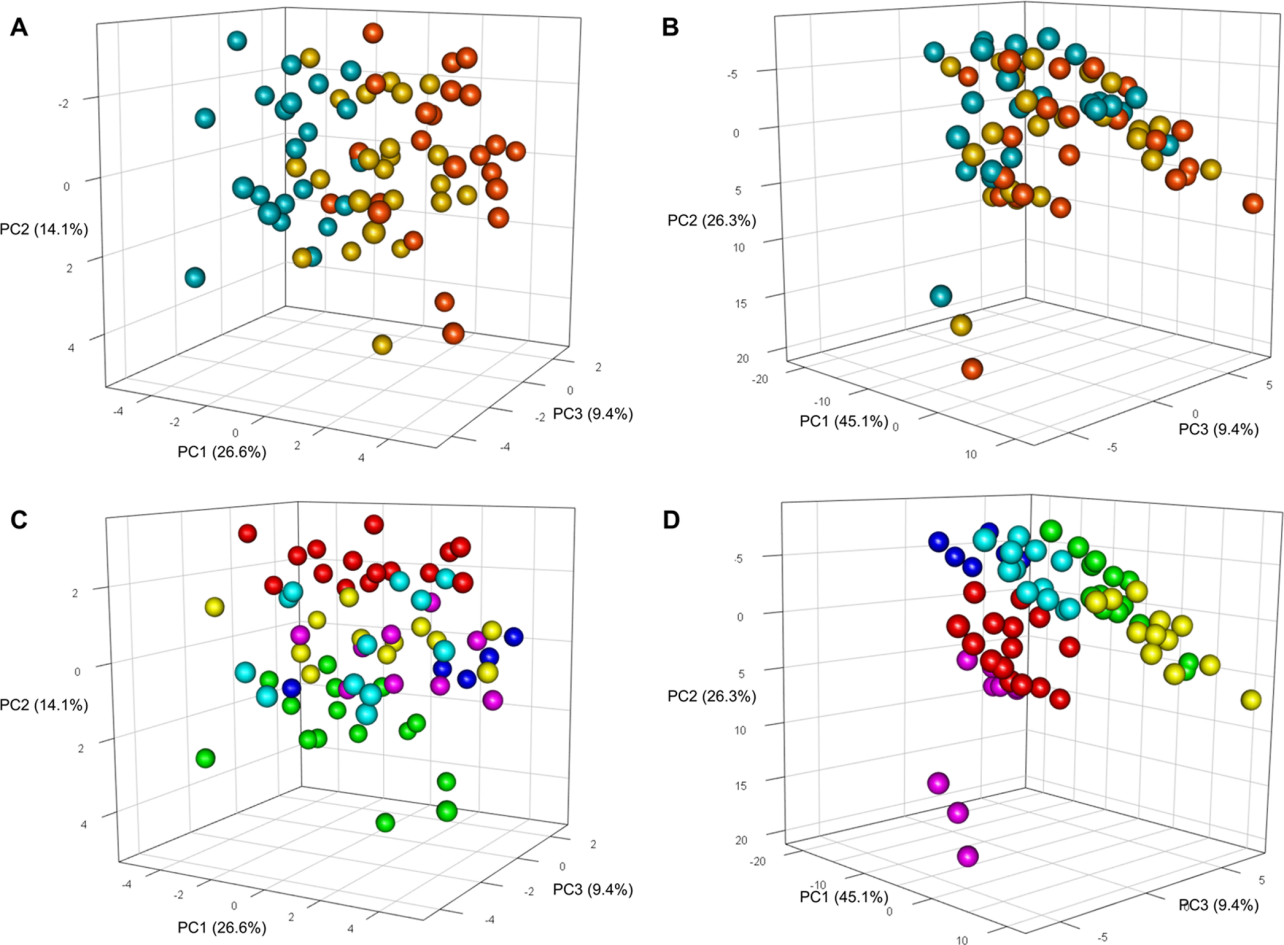
Score plot of the first three principal components of PCA calculated on (A) metabolites and (B) lipoprotein-related parameters. Each sphere represents one NMR sample; spheres are colored according to the blood collection tubes used: serum (red), EDTA plasma (yellow), and citrate plasma (blue). The variance explained by each PC is reported. In (C) and (D), spheres of the same PCA are colored according to the donors (see Figure 1 for color coding).

Unsupervised principal component analysis shows a clear clustering of blood samples based on the collection tubes (Figure 4A), implying that relevant deregulation of metabolites’ levels takes place when different vacutainers are used. In particular, citrate plasma and serum samples present the most striking differences, whereas EDTA plasma samples show an intermediate behavior.

**Figure 5:**
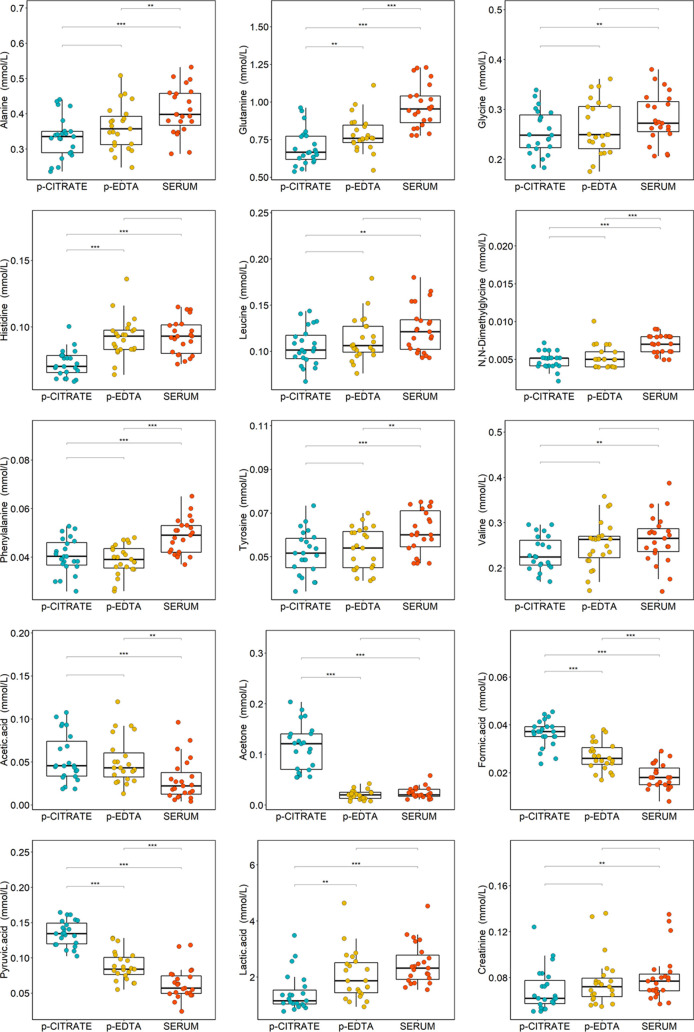

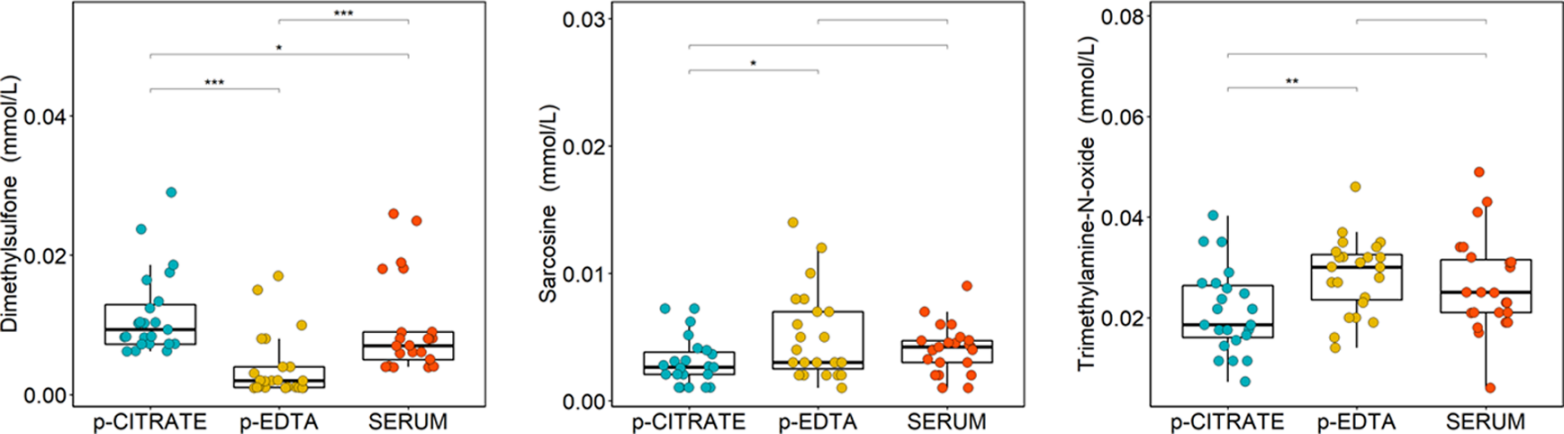
Boxplots of the statistically significant metabolites discriminating serum (red), EDTA plasma (yellow), and citrate plasma (blue) samples; *p*-values adjusted for FDR are reported: ***p < 0.001; **p < 0.01; *p < 0.05.

In detail, with univariate analysis, we found that 18 out of 24 quantified metabolites exhibited statistically different concentrations among citrate plasma, EDTA plasma and serum (Figure 5 and Table 1). As a general trend, amino acids tend to have higher levels in serum samples compared to both plasma samples; in particular, in serum, we observed statistically higher concentrations of alanine, glutamine, glycine, histidine, leucine, N,N-dimethylglycine, phenylalanine, tyrosine, and valine. This evidence is in agreement with previous studies, even across different analytical platforms,^1,5,10,13,16^ and probably resulted from a combination of two factors. First, both anticoagulants have partial inhibitory effects on plasma proteolytic activities^26^ and, second, during the coagulation step of serum sample collection, some metabolites were probably released by activated platelets, increasing their levels.^5^

In serum as compared with citrate and EDTA plasma samples, higher levels of lactate coupled with reduced levels of pyruvate are observed (Figure 5). This phenomenon has been tentatively ascribed to ongoing glycolysis in the time frame of clotting, prior to the separation of serum from the blood cells.^14,27^ Acetone, acetic acid, and formic acid are also shown to be reduced in serum samples, and this is consistent with previous findings.^7,15,16,28^ Pinto et al.^12^ reported that acetic and formic acid are present as contaminants in EDTA plasma tubes. Regarding acetone, Barton et al. proposed it to be due to the contamination introduced at the blood aliquoting stage.^15^ However, we suspected that acetone, acetic acid, and formic acid are released from the plastic or present in the anticoagulant solution of the citrate plasma collection tubes.

Furthermore, we also observed reduced levels of creatinine in citrate plasma compared to serum. EDTA plasma samples display higher levels of trimethylamine-N-oxide and sarcosine compared with citrate plasma. Moreover, dimethylsulfone shows significantly different levels in all of the three blood collection tubes analyzed.

**Figure 6:**
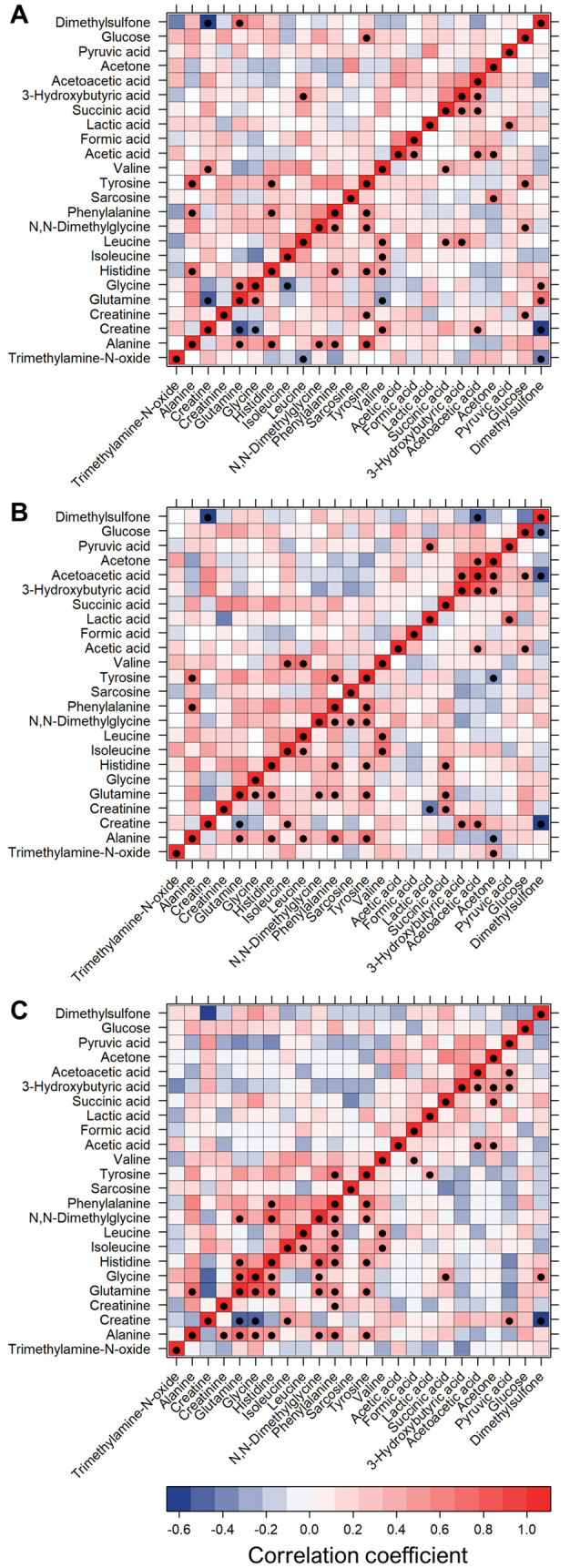
Heatmap showing correlations among metabolites in the three blood derivatives: (A) citrate plasma, (B) EDTA plasma, and (C) serum. *R* values are shown as different degrees of color intensity (red, positive correlations; blue, negative correlation); ● indicates statistically significant correlations (*p*-value < 0.05, up diagonal *p*-values adjusted with FDR correction).

The analysis of the citrate plasma, EDTA plasma, and serum correlation patterns (Figure 6) highlights the presence of several statistically significant differences. Succinic, acetoacetic, and 3-hydroxybutyric acids are strongly correlated in both plasma samples, whereas these relations are partially lost in serum samples. Leucine significantly anticorrelates with 3-hydroxybutyrate in citrate plasma, instead no correlation is present in EDTA plasma and serum. Glutamine, glycine, and histidine show strong positive correlations in serum; however, they are partially lost in both plasma samples.

We also examined the integrals of the region of glycoproteins, which comprises both the NMR signals for GlycA and GlycB (Figure S2). Both GlycA and GlycB were shown to be significantly reduced in citrate plasma samples compared with both EDTA plasma and serum samples. Moreover, GlycA also presents a statistically significant reduction in EDTA plasma compared with serum samples. GlycA and GlycB are biomarkers strongly associated with inflammation and seem to predict future cardiovascular events;^29^ therefore, the phenomenon that we have observed should be taken into consideration when these glycoproteins have to be measured.

### 3.2 Influences of Different Blood collection Tubes on Lipoproteins

Even the lipoproteins’ profiles were shown to be impacted by blood collection tubes; indeed, each triplet of blood-derived samples is more or less distant in the PCA. However, a clear clusterization of blood samples in the PCA space based on the collection tubes, as seen for metabolites, is not visible (Figure 4B), implying that alterations are less marked than those present in the metabolomic profiles. Nine samples (3 citrate plasma, 3 EDTA plasma, and 3 serum samples) were shown to be outliers: two triplets of citrate plasma, EDTA plasma, and serum samples (collected at baseline and after 2 weeks) come from donor number 5 (Figure 4, blue spheres), who displays high levels of several VLDL lipoprotein-related parameters and low levels of several HDL-related parameters. The other triplet of samples (Figure 4, magenta spheres) is associated with the baseline samples of donor number 6, who presents particularly high levels of LDL cholesterol-related parameters. Of note, both donors are males.

**Figure 7:**
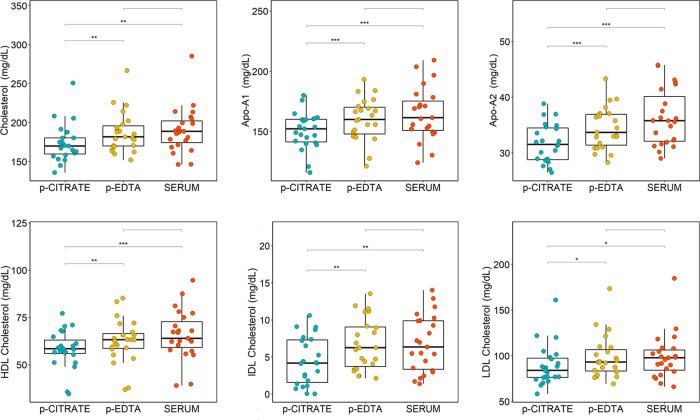
Boxplots of the statistically significant lipoprotein-related parameters (only main parameters) discriminating serum (red), EDTA plasma (yellow), and citrate plasma (blue) samples; *p*-values adjusted for FDR are reported: ***p < 0.001; **p < 0.01; *p < 0.05.

From univariate analysis, it emerged that citrate plasma samples, in general, present reduced lipoprotein levels in comparison to both EDTA plasma and serum samples (Figure 7). In particular, 46 out of the 112 lipoprotein-related parameters were significantly reduced and 5 parameters significantly increased (triglycerides LDL-3, cholesterol VLDL-5, free cholesterol VLDL-5, phospholipids VLDL-5, triglycerides VLDL-5) in citrate plasma samples compared with serum (Table S2). Out of these 51 lipoprotein-related parameters, 42 were shown to be also significantly altered in the comparison between citrate and EDTA plasma; moreover, other six fractions (particle number LDL-4, cholesterol LDL-4, cholesterol LDL-5, free cholesterol LDL-4, phospholipids LDL-5, Apo-B LDL-4) were significantly higher in EDTA samples in comparison to citrate plasma samples (Table S2). Conversely, EDTA plasma samples compared with serum samples exhibit the reduction of only five lipoprotein-related parameters (free cholesterol LDL-2, cholesterol HDL-1, free cholesterol HDL-1, phospholipids HDL-1, Apo-A1 HDL-1).

The correlation patterns of lipoprotein-related parameters (Figure S1) do not present any significant alterations for blood collected with the three different tubes; thus, we can speculate that the reduction of plasma lipoproteins occurs in a concerted manner and does not significantly alter the interrelationship among lipoproteins and, consequently, the biochemistry of the blood samples. Therefore, our data corroborate the evidence that blood samples collected from the same subjects at the same time and place but using different collection tubes provide similar lipoproteomic profiles. Nevertheless, plasma lipoprotein levels were generally lower than those in serum, and it was hypothesized that water could move osmotically from the blood cells into the plasma proving a sort of dilution effect that is more marked in citrate plasma samples in which anticoagulant is present at higher concentrations.^17,30,31^ However, it has been also demonstrated that the lipoprotein composition of plasma extracellular vesicles (EV) significantly differs between plasma and serum, and between anticoagulants.^32^ This phenomenon certainly plays a role in the lipoprotein alterations that we detected, and since EVs transport also metabolites, it is possible to hypothesize its involvement also in the metabolomic differences observed among the different blood derivatives.

### 3.3 Reducing Serum-Plasma Differences via Statistics

**Figure 8:**
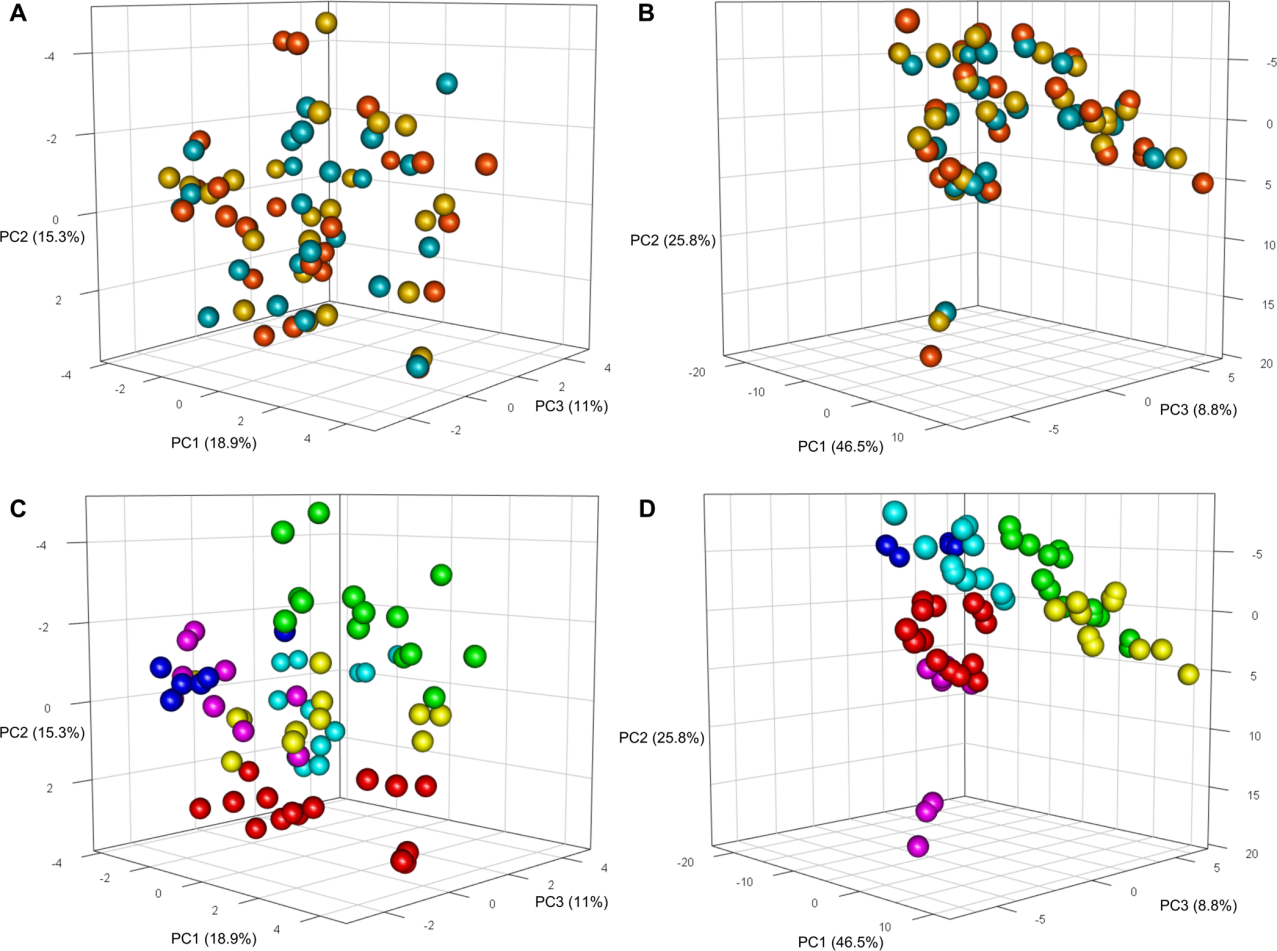
Score plot of the first three principal components of PCA calculated on (A) residuals of metabolites and (B) residuals of lipoprotein-related parameters. Each sphere represents one NMR sample; spheres are colored according to the blood collection tubes used: serum (red), EDTA plasma (yellow), and citrate plasma (blue). The variance explained by each PC is reported. In (C) and (D), spheres of the same PCA are colored according to the donors (see Figure 1 for color coding).

When in a metabolomic and lipoproteomic study different blood matrices have to be compared for statistical analyses, the issue associated with different blood collection tubes became pivotal. Here, we propose a statistical solution for this problem by analyzing the matrix of residuals, obtained via a regression between the categorical variables indicating the collection tubes (category “citrate plasma” encoded as value 1, category “EDTA plasma” encoded as value 2, category “serum” encoded as value 3) and the continuous variables of the concentrations of metabolites or lipoproteins. Our approach is shown to be efficient in reducing the PCA space separation induced by the different collection tubes for both the metabolomic and lipoproteomic profiles (Figure 8A,B): the three groups clustered in the original PCAs show Calinski–Harabasz indices of 16.1 for metabolites and 1.4 for lipoproteins, and the CH values decreased to 4.9 × 10^–32^ and 1.5 × 10^–31^, respectively, in the PCAs calculated on residuals. If we color the PCA according to the donors, we can observe that each subject continued to cluster in her/his PCA subspace (Figure 8C,D). Thus, we may hypothesize that although our approach induced strong alterations in the original data, their biological and physiological properties are conserved.

## 4 Conclusions

The choice of the blood collection matrix is critical to obtain meaningful biological inferences from metabolomic and lipoproteomic data. Our data highlight how strong and different are the alterations and the biochemical reactions that take place when different blood collection tubes are used. Plasma and serum samples not merely differ for the NMR peaks of anticoagulants, but most metabolites proved to have different interrelationships in the different matrices. Some differences are visible also in the lipoproteins’ profile, although less marked. Therefore, the use of different collection tubes, even among the same blood matrix, should be avoided when planning prospective studies and carefully considered in multicenter or retrospective studies when samples are obtained from different centers using different blood collection tubes.

## Supporting Information Available

Data completeness for the different metabolites quantified in the serum samples analyzed via NMR (Table S1); blood lipoprotein-related parameters (ratios, fractions, and subfractions) quantified via ^1^H NMR spectroscopy (Table S2); heatmap showing correlations among lipoprotein-related parameters in the three blood derivatives: citrate plasma, EDTA plasma, and serum (Figure S1); and boxplots of the integrated region of glycoproteins GlycA and GlycB (Figure S2) (PDF)
