# Supplementary material for: Serum or Plasma (and Which Plasma), That Is the Question
Source: J Proteome Res. 2022 Mar 10;21(4):1061–72. doi: 10.1021/acs.jproteome.1c00935 (PMC8981325; doi:10.1021/acs.jproteome.1c00935)
Supplement: Supplementary file 1 — pr1c00935_si_001.pdf [file pr1c00935_si_001.pdf]

Serum or plasma (and which plasma), that is the question

*Alessia Vignoli[1,2], Leonardo Tenori[1,2,3], Cristina Morsiani[4], Paola Turano[1,2,3], Miriam Capri[4], Claudio Luchinat[1,2,3,]**

[1] *Magnetic Resonance Center (CERM), University of Florence, 50019, Sesto Fiorentino, Italy;*

[2] *Department of Chemistry "Ugo Schiff", University of Florence, 50019, Sesto Fiorentino, Italy;*

[3] *Consorzio Interuniversitario Risonanze Magnetiche MetalloProteine (CIRMMP), 50019, Sesto Fiorentino, Italy*

[4] *DIMES-Department of Experimental, Diagnostic and Specialty Medicine, University of Bologna, 40126, Bologna, Italy*

** Corresponding Author, claudioluchinat@cerm.unifi.it*

Supporting Information: Table of Contents

- Table S1. Data completeness for the different metabolites quantified in the serum samples analyzed via NMR.
- Table S2. Blood lipoprotein-related parameters (ratios, fractions and subfractions) quantified via ^{1}H NMR spectroscopy.
- Figure S1. Heatmap showing correlations among lipoprotein-related parameters in the three blood derivatives: A) CITRATE plasma, B) EDTA plasma, C) serum.
- Figure S2. Boxplots of the integrated region of glycoproteins A) GlycA; B) GlycB.

Table S1. Data completeness for the different metabolites quantified in the serum samples analyzed via NMR. LOQ = limit of quantification.

	Results outside the measurable range (%)	***LOQ (mmol/L)***	***95% Range (mmol/L)***
3-Hydroxybutyric acid	21.7	< 0.02	≤ 0.26
Acetic acid	0.0	< 0.01	≤ 0.06
Acetoacetic acid	1.4	< 0.01	≤ 0.02
Acetone	1.4	< 0.01	≤ 0.06
Alanine	0.0	< 0.02	0.64 - 0.29
Asparagine	88.4	< 0.05	≤ 0.08
Choline	89.9	< 0.05	≤ 0.06
Creatine	0.0	< 0.01	≤ 0.07
Creatinine	0.0	< 0.01	0.14 - 0.06
Dimethylsulfone	0.0	< 0.01	≤ 0.02
Ethanol	98.6	< 0.1	≤ 0.82
Formic acid	0.0	< 0.02	≤ 0.03
Glucose	0.0	< 0.54	6.08 - 1.73
Glutamic acid	98.6	< 0.05	≤ 0.24
Glutamine	0.0	< 0.02	0.83 - 0.3
Glycine	0.0	< 0.01	0.44 - 0.17
Histidine	0.0	< 0.02	0.16 - 0.07
Isoleucine	0.0	< 0.03	0.11 - 0.03
Lactic acid	0.0	< 0.03	7.14 - 2.23
Leucine	0.0	< 0.01	0.2 - 0.07
Lysine	65.2	< 0.04	≤ 0.29
Methionine	43.5	< 0.05	0.05 - 0.13
N,N-Dimethylglycine	4.3	< 0.01	≤ 0.01
Ornithine	71.0	< 0.02	≤ 0.16
Phenylalanine	0.0	< 0.03	≤ 0.07
Proline	87.0	< 0.05	≤ 0.59
Pyruvic acid	0.0	< 0.03	≤ 0.07
Sarcosine	23.2	< 0.01	≤ 0.01
Succinic acid	8.7	< 0.01	≤ 0.01
Threonine	26.1	< 0.04	≤ 0.24
Trimethylamine-N-oxide	0.0	< 0.08	≤ 0.08
Tyrosine	0.0	< 0.03	≤ 0.08
Valine	0.0	< 0.03	0.35 - 0.15

Table S2. Blood lipoprotein-related parameters (ratios, fractions and subfractions) quantified via ^{1}H NMR spectroscopy.

	CITRATE Plasma	EDTA Plasma	SERUM	p-value citrate-EDTA	p-value citrate-serum	p-value EDTA-serum
**Ratios**						
LDL-Chol/HDL-Chol						
Mean (SD)	$1.66\cdot10^{0}$ ($5.98\cdot10^{-1}$)	$1.66\cdot10^{0}$ ($5.93\cdot10^{-1}$)	$1.58\cdot10^{0}$ ($5.66\cdot10^{-1}$)	$9.54\cdot10^{-1}$	$8.37\cdot10^{-2}$	$1.93\cdot10^{-1}$
95% CI	($1.40\cdot10^{0}$-$1.92\cdot10^{0}$)	($1.40\cdot10^{0}$-$1.91\cdot10^{0}$)	($1.33\cdot10^{0}$-$1.82\cdot10^{0}$)			
Apo-B100/Apo-A1						
Mean (SD)	$4.53\cdot10^{-1}$ ($1.28\cdot10^{-1}$)	$4.36\cdot10^{-1}$ ($1.28\cdot10^{-1}$)	$4.24\cdot10^{-1}$ ($1.27\cdot10^{-1}$)	$2.45\cdot10^{-2}$	$5.05\cdot10^{-4}$	$2.57\cdot10^{-1}$
95% CI	($3.98\cdot10^{-1}$-$5.09\cdot10^{-1}$)	($3.80\cdot10^{-1}$-$4.91\cdot10^{-1}$)	($3.69\cdot10^{-1}$-$4.79\cdot10^{-1}$)			
**Particle numbers (nmol/L)**						
Total ApoB Particle Number						
Mean (SD)	$1.18\cdot10^{3}$ ($2.65\cdot10^{2}$)	$1.24\cdot10^{3}$ ($2.80\cdot10^{2}$)	$1.24\cdot10^{3}$ ($2.85\cdot10^{2}$)	$5.07\cdot10^{-2}$	$1.06\cdot10^{-1}$	$9.95\cdot10^{-1}$
95% CI	($1.06\cdot10^{3}$-$1.29\cdot10^{3}$)	($1.12\cdot10^{3}$-$1.36\cdot10^{3}$)	($1.12\cdot10^{3}$-$1.36\cdot10^{3}$)			
VLDL Particle Number						
Mean (SD)	$8.13\cdot10^{1}$ ($3.33\cdot10^{1}$)	$7.74\cdot10^{1}$ ($3.55\cdot10^{1}$)	$7.68\cdot10^{1}$ ($3.84\cdot10^{1}$)	$6.54\cdot10^{-1}$	$5.53\cdot10^{-1}$	$9.95\cdot10^{-1}$
95% CI	($6.69\cdot10^{1}$-$9.57\cdot10^{1}$)	($6.20\cdot10^{1}$-$9.27\cdot10^{1}$)	($6.02\cdot10^{1}$-$9.33\cdot10^{1}$)			
IDL Particle Number						
Mean (SD)	$4.09\cdot10^{1}$ ($1.92\cdot10^{1}$)	$5.42\cdot10^{1}$ ($2.05\cdot10^{1}$)	$6.11\cdot10^{1}$ ($2.36\cdot10^{1}$)	$1.56\cdot10^{-3}$	$4.68\cdot10^{-5}$	$2.50\cdot10^{-1}$
95% CI	($3.25\cdot10^{1}$-$4.92\cdot10^{1}$)	($4.53\cdot10^{1}$-$6.31\cdot10^{1}$)	($5.09\cdot10^{1}$-$7.13\cdot10^{1}$)			
LDL Particle Number						
Mean (SD)	$1.05\cdot10^{3}$ ($2.39\cdot10^{2}$)	$1.12\cdot10^{3}$ ($2.53\cdot10^{2}$)	$1.12\cdot10^{3}$ ($2.60\cdot10^{2}$)	$6.57\cdot10^{-2}$	$1.06\cdot10^{-1}$	$9.95\cdot10^{-1}$
95% CI	($9.43\cdot10^{2}$-$1.15\cdot10^{3}$)	($1.01\cdot10^{3}$-$1.23\cdot10^{3}$)	($1.00\cdot10^{3}$-$1.23\cdot10^{3}$)			
LDL-1 Particle Number						
Mean (SD)	$2.06\cdot10^{2}$ ($3.80\cdot10^{1}$)	$2.05\cdot10^{2}$ ($4.01\cdot10^{1}$)	$2.17\cdot10^{2}$ ($4.56\cdot10^{1}$)	$8.70\cdot10^{-1}$	$2.00\cdot10^{-1}$	$2.50\cdot10^{-1}$
95% CI	($1.90\cdot10^{2}$-$2.23\cdot10^{2}$)	($1.88\cdot10^{2}$-$2.22\cdot10^{2}$)	($1.98\cdot10^{2}$-$2.37\cdot10^{2}$)			
LDL-2 Particle Number						
Mean (SD)	$1.45\cdot10^{2}$ ($4.39\cdot10^{1}$)	$1.48\cdot10^{2}$ ($4.56\cdot10^{1}$)	$1.67\cdot10^{2}$ ($5.46\cdot10^{1}$)	$7.13\cdot10^{-1}$	$9.61\cdot10^{-3}$	$8.16\cdot10^{-2}$
95% CI	($1.26\cdot10^{2}$-$1.64\cdot10^{2}$)	($1.28\cdot10^{2}$-$1.68\cdot10^{2}$)	($1.44\cdot10^{2}$-$1.91\cdot10^{2}$)			
LDL-3 Particle Number						
Mean (SD)	$1.63\cdot10^{2}$ ($5.09\cdot10^{1}$)	$1.77\cdot10^{2}$ ($5.56\cdot10^{1}$)	$1.76\cdot10^{2}$ ($6.14\cdot10^{1}$)	$1.35\cdot10^{-1}$	$2.31\cdot10^{-1}$	$9.95\cdot10^{-1}$
95% CI	($1.41\cdot10^{2}$-$1.85\cdot10^{2}$)	($1.53\cdot10^{2}$-$2.01\cdot10^{2}$)	($1.49\cdot10^{2}$-$2.02\cdot10^{2}$)			
LDL-4 Particle Number						
Mean (SD)	$1.48\cdot10^{2}$ ($7.23\cdot10^{1}$)	$1.72\cdot10^{2}$ ($7.77\cdot10^{1}$)	$1.52\cdot10^{2}$ ($7.99\cdot10^{1}$)	$4.07\cdot10^{-2}$	$8.00\cdot10^{-1}$	$2.50\cdot10^{-1}$
95% CI	($1.17\cdot10^{2}$-$1.79\cdot10^{2}$)	($1.38\cdot10^{2}$-$2.06\cdot10^{2}$)	($1.17\cdot10^{2}$-$1.86\cdot10^{2}$)			
LDL-5 Particle Number						
Mean (SD)	$1.15\cdot10^{2}$ ($7.51\cdot10^{1}$)	$1.42\cdot10^{2}$ ($7.96\cdot10^{1}$)	$1.37\cdot10^{2}$ ($8.06\cdot10^{1}$)	$1.33\cdot10^{-3}$	$2.85\cdot10^{-2}$	$9.36\cdot10^{-1}$
95% CI	($8.29\cdot10^{1}$-$1.48\cdot10^{2}$)	($1.08\cdot10^{2}$-$1.77\cdot10^{2}$)	($1.02\cdot10^{2}$-$1.72\cdot10^{2}$)			
LDL-6 Particle Number						
Mean (SD)	$2.46\cdot10^{2}$ ($8.50\cdot10^{1}$)	$2.66\cdot10^{2}$ ($9.17\cdot10^{1}$)	$2.68\cdot10^{2}$ ($9.03\cdot10^{1}$)	$5.23\cdot10^{-2}$	$4.93\cdot10^{-2}$	$9.95\cdot10^{-1}$
95% CI	($2.09\cdot10^{2}$-$2.82\cdot10^{2}$)	($2.27\cdot10^{2}$-$3.06\cdot10^{2}$)	($2.29\cdot10^{2}$-$3.07\cdot10^{2}$)			
**Lipoprotein parameters (mg/dL)**						
Triglycerides, VLDL						
Mean (SD)	$3.42\cdot10^{1}$ ($2.39\cdot10^{1}$)	$3.75\cdot10^{1}$ ($2.56\cdot10^{1}$)	$3.72\cdot10^{1}$ ($2.64\cdot10^{1}$)	$4.90\cdot10^{-1}$	$4.81\cdot10^{-1}$	$9.95\cdot10^{-1}$
95% CI	($2.38\cdot10^{1}$-$4.45\cdot10^{1}$)	($2.64\cdot10^{1}$-$4.85\cdot10^{1}$)	($2.58\cdot10^{1}$-$4.86\cdot10^{1}$)			
Triglycerides, IDL						
Mean (SD)	$3.39\cdot10^{0}$ ($4.08\cdot10^{0}$)	$4.08\cdot10^{0}$ ($4.38\cdot10^{0}$)	$4.57\cdot10^{0}$ ($4.53\cdot10^{0}$)	$3.32\cdot10^{-1}$	$8.47\cdot10^{-2}$	$8.54\cdot10^{-1}$
95% CI	($1.63\cdot10^{0}$-$5.16\cdot10^{0}$)	($2.19\cdot10^{0}$-$5.98\cdot10^{0}$)	($2.61\cdot10^{0}$-$6.52\cdot10^{0}$)			
Triglycerides, LDL						
Mean (SD)	$1.39\cdot10^{1}$ ($2.19\cdot10^{0}$)	$1.44\cdot10^{1}$ ($2.25\cdot10^{0}$)	$1.47\cdot10^{1}$ ($2.61\cdot10^{0}$)	$3.74\cdot10^{-1}$	$1.39\cdot10^{-1}$	$9.09\cdot10^{-1}$
95% CI	($1.30\cdot10^{1}$-$1.49\cdot10^{1}$)	($1.34\cdot10^{1}$-$1.54\cdot10^{1}$)	($1.36\cdot10^{1}$-$1.58\cdot10^{1}$)			

Triglycerides, HDL						
Mean (SD)<br>95% CI	$7.43\cdot10^{0}$ ($1.44\cdot10^{0}$)<br>($6.81\cdot10^{0}$-$8.05\cdot10^{0}$)	$7.48\cdot10^{0}$ ($1.61\cdot10^{0}$)<br>($6.78\cdot10^{0}$-$8.17\cdot10^{0}$)	$7.32\cdot10^{0}$ ($1.69\cdot10^{0}$)<br>($6.59\cdot10^{0}$-$8.06\cdot10^{0}$)	$8.97\cdot10^{-1}$	$8.25\cdot10^{-1}$	$9.93\cdot10^{-1}$
Free Cholesterol, VLDL						
Mean (SD)<br>95% CI	$5.16\cdot10^{0}$ ($2.53\cdot10^{0}$)<br>($4.07\cdot10^{0}$-$6.25\cdot10^{0}$)	$4.96\cdot10^{0}$ ($2.68\cdot10^{0}$)<br>($3.80\cdot10^{0}$-$6.12\cdot10^{0}$)	$4.57\cdot10^{0}$ ($2.91\cdot10^{0}$)<br>($3.31\cdot10^{0}$-$5.82\cdot10^{0}$)	$7.13\cdot10^{-1}$	$2.51\cdot10^{-1}$	$7.97\cdot10^{-1}$
Free Cholesterol, IDL						
Mean (SD)<br>95% CI	$1.33\cdot10^{0}$ ($9.69\cdot10^{-1}$)<br>($9.12\cdot10^{-1}$-$1.75\cdot10^{0}$)	$1.96\cdot10^{0}$ ($1.03\cdot10^{0}$)<br>($1.52\cdot10^{0}$-$2.41\cdot10^{0}$)	$2.06\cdot10^{0}$ ($1.14\cdot10^{0}$)<br>($1.57\cdot10^{0}$-$2.55\cdot10^{0}$)	$1.33\cdot10^{-3}$	$6.70\cdot10^{-4}$	$9.93\cdot10^{-1}$
Free Cholesterol, LDL						
Mean (SD)<br>95% CI	$2.75\cdot10^{1}$ ($5.78\cdot10^{0}$)<br>($2.50\cdot10^{1}$-$3.00\cdot10^{1}$)	$2.97\cdot10^{1}$ ($6.22\cdot10^{0}$)<br>($2.70\cdot10^{1}$-$3.24\cdot10^{1}$)	$3.05\cdot10^{1}$ ($6.59\cdot10^{0}$)<br>($2.76\cdot10^{1}$-$3.33\cdot10^{1}$)	$2.89\cdot10^{-2}$	$1.29\cdot10^{-2}$	$9.09\cdot10^{-1}$
Free Cholesterol, HDL						
Mean (SD)<br>95% CI	$1.32\cdot10^{1}$ ($2.65\cdot10^{0}$)<br>($1.21\cdot10^{1}$-$1.43\cdot10^{1}$)	$1.40\cdot10^{1}$ ($2.98\cdot10^{0}$)<br>($1.28\cdot10^{1}$-$1.53\cdot10^{1}$)	$1.47\cdot10^{1}$ ($3.32\cdot10^{0}$)<br>($1.32\cdot10^{1}$-$1.61\cdot10^{1}$)	$8.39\cdot10^{-3}$	$2.17\cdot10^{-4}$	$2.31\cdot10^{-1}$
Phospholipids, VLDL						
Mean (SD)<br>95% CI	$1.08\cdot10^{1}$ ($6.04\cdot10^{0}$)<br>($8.14\cdot10^{0}$-$1.34\cdot10^{1}$)	$1.05\cdot10^{1}$ ($6.43\cdot10^{0}$)<br>($7.75\cdot10^{0}$-$1.33\cdot10^{1}$)	$9.97\cdot10^{0}$ ($6.78\cdot10^{0}$)<br>($7.04\cdot10^{0}$-$1.29\cdot10^{1}$)	$8.54\cdot10^{-1}$	$5.02\cdot10^{-1}$	$9.93\cdot10^{-1}$
Phospholipids, IDL						
Mean (SD)<br>95% CI	$2.66\cdot10^{0}$ ($1.99\cdot10^{0}$)<br>($1.80\cdot10^{0}$-$3.52\cdot10^{0}$)	$4.08\cdot10^{0}$ ($2.10\cdot10^{0}$)<br>($3.17\cdot10^{0}$-$4.99\cdot10^{0}$)	$4.85\cdot10^{0}$ ($2.26\cdot10^{0}$)<br>($3.88\cdot10^{0}$-$5.83\cdot10^{0}$)	$4.44\cdot10^{-4}$	$2.91\cdot10^{-6}$	$1.69\cdot10^{-1}$
Phospholipids, LDL						
Mean (SD)<br>95% CI	$5.38\cdot10^{1}$ ($1.05\cdot10^{1}$)<br>($4.92\cdot10^{1}$-$5.84\cdot10^{1}$)	$5.74\cdot10^{1}$ ($1.11\cdot10^{1}$)<br>($5.26\cdot10^{1}$-$6.22\cdot10^{1}$)	$5.76\cdot10^{1}$ ($1.19\cdot10^{1}$)<br>($5.25\cdot10^{1}$-$6.27\cdot10^{1}$)	$5.07\cdot10^{-2}$	$8.37\cdot10^{-2}$	$9.95\cdot10^{-1}$
Phospholipids, HDL						
Mean (SD)<br>95% CI	$8.33\cdot10^{1}$ ($1.43\cdot10^{1}$)<br>($7.71\cdot10^{1}$-$8.95\cdot10^{1}$)	$8.79\cdot10^{1}$ ($1.62\cdot10^{1}$)<br>($8.09\cdot10^{1}$-$9.49\cdot10^{1}$)	$9.20\cdot10^{1}$ ($1.80\cdot10^{1}$)<br>($8.42\cdot10^{1}$-$9.98\cdot10^{1}$)	$7.31\cdot10^{-3}$	$7.97\cdot10^{-5}$	$1.28\cdot10^{-1}$
Apo-A1, HDL						
Mean (SD)<br>95% CI	$1.49\cdot10^{2}$ ($1.67\cdot10^{1}$)<br>($1.42\cdot10^{2}$-$1.56\cdot10^{2}$)	$1.58\cdot10^{2}$ ($1.91\cdot10^{1}$)<br>($1.50\cdot10^{2}$-$1.66\cdot10^{2}$)	$1.64\cdot10^{2}$ ($2.34\cdot10^{1}$)<br>($1.54\cdot10^{2}$-$1.74\cdot10^{2}$)	$1.33\cdot10^{-3}$	$4.68\cdot10^{-5}$	$1.69\cdot10^{-1}$
Apo-A2, HDL						
Mean (SD)<br>95% CI	$3.21\cdot10^{1}$ ($3.21\cdot10^{0}$)<br>($3.07\cdot10^{1}$-$3.35\cdot10^{1}$)	$3.45\cdot10^{1}$ ($3.52\cdot10^{0}$)<br>($3.30\cdot10^{1}$-$3.61\cdot10^{1}$)	$3.62\cdot10^{1}$ ($4.77\cdot10^{0}$)<br>($3.42\cdot10^{1}$-$3.83\cdot10^{1}$)	$7.68\cdot10^{-4}$	$3.52\cdot10^{-5}$	$1.69\cdot10^{-1}$
Apo-B, VLDL						
Mean (SD)<br>95% CI	$4.47\cdot10^{0}$ ($1.83\cdot10^{0}$)<br>($3.68\cdot10^{0}$-$5.26\cdot10^{0}$)	$4.26\cdot10^{0}$ ($1.95\cdot10^{0}$)<br>($3.41\cdot10^{0}$-$5.10\cdot10^{0}$)	$4.22\cdot10^{0}$ ($2.11\cdot10^{0}$)<br>($3.31\cdot10^{0}$-$5.13\cdot10^{0}$)	$6.54\cdot10^{-1}$	$5.53\cdot10^{-1}$	$9.95\cdot10^{-1}$
Apo-B, IDL						
Mean (SD)<br>95% CI	$2.25\cdot10^{0}$ ($1.06\cdot10^{0}$)<br>($1.79\cdot10^{0}$-$2.71\cdot10^{0}$)	$2.98\cdot10^{0}$ ($1.13\cdot10^{0}$)<br>($2.49\cdot10^{0}$-$3.47\cdot10^{0}$)	$3.36\cdot10^{0}$ ($1.30\cdot10^{0}$)<br>($2.80\cdot10^{0}$-$3.92\cdot10^{0}$)	$1.56\cdot10^{-3}$	$4.68\cdot10^{-5}$	$2.50\cdot10^{-1}$
Apo-B, LDL						
Mean (SD)<br>95% CI	$5.76\cdot10^{1}$ ($1.32\cdot10^{1}$)<br>($5.19\cdot10^{1}$-$6.32\cdot10^{1}$)	$6.14\cdot10^{1}$ ($1.39\cdot10^{1}$)<br>($5.53\cdot10^{1}$-$6.74\cdot10^{1}$)	$6.14\cdot10^{1}$ ($1.43\cdot10^{1}$)<br>($5.52\cdot10^{1}$-$6.76\cdot10^{1}$)	$6.57\cdot10^{-2}$	$1.06\cdot10^{-1}$	$9.95\cdot10^{-1}$
Triglycerides, VLDL-1						
Mean (SD)<br>95% CI	$1.11\cdot10^{1}$ ($1.48\cdot10^{1}$)<br>($4.76\cdot10^{0}$-$1.75\cdot10^{1}$)	$1.25\cdot10^{1}$ ($1.59\cdot10^{1}$)<br>($5.66\cdot10^{0}$-$1.94\cdot10^{1}$)	$1.25\cdot10^{1}$ ($1.64\cdot10^{1}$)<br>($5.42\cdot10^{0}$-$1.96\cdot10^{1}$)	$5.46\cdot10^{-1}$	$4.97\cdot10^{-1}$	$9.95\cdot10^{-1}$
Triglycerides, VLDL-2						
Mean (SD)<br>95% CI	$4.95\cdot10^{0}$ ($4.95\cdot10^{0}$)<br>($2.81\cdot10^{0}$-$7.09\cdot10^{0}$)	$5.63\cdot10^{0}$ ($5.31\cdot10^{0}$)<br>($3.33\cdot10^{0}$-$7.92\cdot10^{0}$)	$5.68\cdot10^{0}$ ($5.55\cdot10^{0}$)<br>($3.28\cdot10^{0}$-$8.08\cdot10^{0}$)	$5.74\cdot10^{-1}$	$4.90\cdot10^{-1}$	$9.95\cdot10^{-1}$
Triglycerides, VLDL-3						
Mean (SD)<br>95% CI	$4.28\cdot10^{0}$ ($3.58\cdot10^{0}$)<br>($2.73\cdot10^{0}$-$5.83\cdot10^{0}$)	$4.72\cdot10^{0}$ ($3.85\cdot10^{0}$)<br>($3.06\cdot10^{0}$-$6.39\cdot10^{0}$)	$4.80\cdot10^{0}$ ($4.04\cdot10^{0}$)<br>($3.05\cdot10^{0}$-$6.55\cdot10^{0}$)	$6.83\cdot10^{-1}$	$5.75\cdot10^{-1}$	$9.95\cdot10^{-1}$
Triglycerides, VLDL-4						
Mean (SD)<br>95% CI	$4.22\cdot10^{0}$ ($2.01\cdot10^{0}$)<br>($3.35\cdot10^{0}$-$5.09\cdot10^{0}$)	$4.49\cdot10^{0}$ ($2.18\cdot10^{0}$)<br>($3.55\cdot10^{0}$-$5.43\cdot10^{0}$)	$4.28\cdot10^{0}$ ($2.29\cdot10^{0}$)<br>($3.29\cdot10^{0}$-$5.27\cdot10^{0}$)	$7.13\cdot10^{-1}$	$9.28\cdot10^{-1}$	$9.93\cdot10^{-1}$
Triglycerides, VLDL-5						

Mean (SD)	$2.56\cdot10^{0}$ ($4.78\cdot10^{-1}$)	$2.29\cdot10^{0}$ ($5.17\cdot10^{-1}$)	$2.05\cdot10^{0}$ ($5.50\cdot10^{-1}$)	$1.33\cdot10^{-2}$	$3.52\cdot10^{-5}$	$1.66\cdot10^{-1}$
95% CI	($2.35\cdot10^{0}$-$2.76\cdot10^{0}$)	($2.06\cdot10^{0}$-$2.51\cdot10^{0}$)	($1.81\cdot10^{0}$-$2.29\cdot10^{0}$)			
Cholesterol, VLDL-1						
Mean (SD)	$3.57\cdot10^{0}$ ($2.51\cdot10^{0}$)	$3.46\cdot10^{0}$ ($2.66\cdot10^{0}$)	$3.31\cdot10^{0}$ ($2.93\cdot10^{0}$)	$8.03\cdot10^{-1}$	$5.36\cdot10^{-1}$	$9.93\cdot10^{-1}$
95% CI	($2.48\cdot10^{0}$-$4.65\cdot10^{0}$)	($2.31\cdot10^{0}$-$4.61\cdot10^{0}$)	($2.05\cdot10^{0}$-$4.58\cdot10^{0}$)			
Cholesterol, VLDL-2						
Mean (SD)	$1.73\cdot10^{0}$ ($1.18\cdot10^{0}$)	$1.79\cdot10^{0}$ ($1.24\cdot10^{0}$)	$1.86\cdot10^{0}$ ($1.37\cdot10^{0}$)	$8.03\cdot10^{-1}$	$5.69\cdot10^{-1}$	$9.93\cdot10^{-1}$
95% CI	($1.22\cdot10^{0}$-$2.24\cdot10^{0}$)	($1.25\cdot10^{0}$-$2.33\cdot10^{0}$)	($1.27\cdot10^{0}$-$2.45\cdot10^{0}$)			
Cholesterol, VLDL-3						
Mean (SD)	$1.18\cdot10^{0}$ ($1.28\cdot10^{0}$)	$1.41\cdot10^{0}$ ($1.38\cdot10^{0}$)	$1.52\cdot10^{0}$ ($1.50\cdot10^{0}$)	$5.55\cdot10^{-1}$	$3.06\cdot10^{-1}$	$9.93\cdot10^{-1}$
95% CI	($6.30\cdot10^{-1}$-$1.73\cdot10^{0}$)	($8.16\cdot10^{-1}$-$2.01\cdot10^{0}$)	($8.70\cdot10^{-1}$-$2.17\cdot10^{0}$)			
Cholesterol, VLDL-4						
Mean (SD)	$2.18\cdot10^{0}$ ($1.29\cdot10^{0}$)	$2.34\cdot10^{0}$ ($1.36\cdot10^{0}$)	$2.35\cdot10^{0}$ ($1.52\cdot10^{0}$)	$6.83\cdot10^{-1}$	$6.14\cdot10^{-1}$	$9.95\cdot10^{-1}$
95% CI	($1.62\cdot10^{0}$-$2.74\cdot10^{0}$)	($1.75\cdot10^{0}$-$2.93\cdot10^{0}$)	($1.70\cdot10^{0}$-$3.01\cdot10^{0}$)			
Cholesterol, VLDL-5						
Mean (SD)	$1.39\cdot10^{0}$ ($3.75\cdot10^{-1}$)	$1.02\cdot10^{0}$ ($4.21\cdot10^{-1}$)	$8.19\cdot10^{-1}$ ($4.66\cdot10^{-1}$)	$3.66\cdot10^{-4}$	$3.56\cdot10^{-6}$	$1.69\cdot10^{-1}$
95% CI	($1.23\cdot10^{0}$-$1.56\cdot10^{0}$)	($8.36\cdot10^{-1}$-$1.20\cdot10^{0}$)	($6.18\cdot10^{-1}$-$1.02\cdot10^{0}$)			
Free Cholesterol, VLDL-1						
Mean (SD)	$9.82\cdot10^{-1}$ ($1.22\cdot10^{0}$)	$1.24\cdot10^{0}$ ($1.33\cdot10^{0}$)	$1.24\cdot10^{0}$ ($1.36\cdot10^{0}$)	$1.35\cdot10^{-1}$	$1.36\cdot10^{-1}$	$9.95\cdot10^{-1}$
95% CI	($4.54\cdot10^{-1}$-$1.51\cdot10^{0}$)	($6.62\cdot10^{-1}$-$1.81\cdot10^{0}$)	($6.51\cdot10^{-1}$-$1.82\cdot10^{0}$)			
Free Cholesterol, VLDL-2						
Mean (SD)	$6.74\cdot10^{-1}$ ($4.83\cdot10^{-1}$)	$7.00\cdot10^{-1}$ ($5.08\cdot10^{-1}$)	$6.58\cdot10^{-1}$ ($5.68\cdot10^{-1}$)	$8.12\cdot10^{-1}$	$8.80\cdot10^{-1}$	$9.93\cdot10^{-1}$
95% CI	($4.65\cdot10^{-1}$-$8.83\cdot10^{-1}$)	($4.80\cdot10^{-1}$-$9.20\cdot10^{-1}$)	($4.12\cdot10^{-1}$-$9.04\cdot10^{-1}$)			
Free Cholesterol, VLDL-3						
Mean (SD)	$4.52\cdot10^{-1}$ ($5.40\cdot10^{-1}$)	$5.39\cdot10^{-1}$ ($5.88\cdot10^{-1}$)	$5.86\cdot10^{-1}$ ($6.34\cdot10^{-1}$)	$6.14\cdot10^{-1}$	$3.56\cdot10^{-1}$	$9.93\cdot10^{-1}$
95% CI	($2.18\cdot10^{-1}$-$6.85\cdot10^{-1}$)	($2.85\cdot10^{-1}$-$7.93\cdot10^{-1}$)	($3.12\cdot10^{-1}$-$8.60\cdot10^{-1}$)			
Free Cholesterol, VLDL-4						
Mean (SD)	$6.86\cdot10^{-1}$ ($5.29\cdot10^{-1}$)	$9.37\cdot10^{-1}$ ($5.86\cdot10^{-1}$)	$9.18\cdot10^{-1}$ ($6.15\cdot10^{-1}$)	$1.08\cdot10^{-1}$	$1.36\cdot10^{-1}$	$9.95\cdot10^{-1}$
95% CI	($4.57\cdot10^{-1}$-$9.15\cdot10^{-1}$)	($6.83\cdot10^{-1}$-$1.19\cdot10^{0}$)	($6.52\cdot10^{-1}$-$1.19\cdot10^{0}$)			
Free Cholesterol, VLDL-5						
Mean (SD)	$5.35\cdot10^{-1}$ ($2.26\cdot10^{-1}$)	$2.85\cdot10^{-1}$ ($2.24\cdot10^{-1}$)	$2.70\cdot10^{-1}$ ($2.36\cdot10^{-1}$)	$8.72\cdot10^{-5}$	$1.22\cdot10^{-5}$	$9.95\cdot10^{-1}$
95% CI	($4.38\cdot10^{-1}$-$6.33\cdot10^{-1}$)	($1.88\cdot10^{-1}$-$3.82\cdot10^{-1}$)	($1.68\cdot10^{-1}$-$3.72\cdot10^{-1}$)			
Phospholipids, VLDL-1						
Mean (SD)	$2.09\cdot10^{0}$ ($2.63\cdot10^{0}$)	$2.34\cdot10^{0}$ ($2.81\cdot10^{0}$)	$2.32\cdot10^{0}$ ($2.93\cdot10^{0}$)	$5.65\cdot10^{-1}$	$5.41\cdot10^{-1}$	$9.95\cdot10^{-1}$
95% CI	($9.55\cdot10^{-1}$-$3.23\cdot10^{0}$)	($1.12\cdot10^{0}$-$3.56\cdot10^{0}$)	($1.05\cdot10^{0}$-$3.58\cdot10^{0}$)			
Phospholipids, VLDL-2						
Mean (SD)	$1.50\cdot10^{0}$ ($1.33\cdot10^{0}$)	$1.67\cdot10^{0}$ ($1.42\cdot10^{0}$)	$1.67\cdot10^{0}$ ($1.49\cdot10^{0}$)	$5.89\cdot10^{-1}$	$5.02\cdot10^{-1}$	$9.95\cdot10^{-1}$
95% CI	($9.21\cdot10^{-1}$-$2.08\cdot10^{0}$)	($1.05\cdot10^{0}$-$2.28\cdot10^{0}$)	($1.03\cdot10^{0}$-$2.32\cdot10^{0}$)			
Phospholipids, VLDL-3						
Mean (SD)	$1.44\cdot10^{0}$ ($1.23\cdot10^{0}$)	$1.77\cdot10^{0}$ ($1.32\cdot10^{0}$)	$1.86\cdot10^{0}$ ($1.39\cdot10^{0}$)	$2.86\cdot10^{-1}$	$1.56\cdot10^{-1}$	$9.93\cdot10^{-1}$
95% CI	($9.03\cdot10^{-1}$-$1.97\cdot10^{0}$)	($1.20\cdot10^{0}$-$2.34\cdot10^{0}$)	($1.26\cdot10^{0}$-$2.46\cdot10^{0}$)			
Phospholipids, VLDL-4						
Mean (SD)	$2.38\cdot10^{0}$ ($9.84\cdot10^{-1}$)	$2.51\cdot10^{0}$ ($1.05\cdot10^{0}$)	$2.39\cdot10^{0}$ ($1.13\cdot10^{0}$)	$6.75\cdot10^{-1}$	$9.96\cdot10^{-1}$	$9.93\cdot10^{-1}$
95% CI	($1.96\cdot10^{0}$-$2.81\cdot10^{0}$)	($2.06\cdot10^{0}$-$2.97\cdot10^{0}$)	($1.90\cdot10^{0}$-$2.88\cdot10^{0}$)			
Phospholipids, VLDL-5						
Mean (SD)	$1.57\cdot10^{0}$ ($4.06\cdot10^{-1}$)	$1.23\cdot10^{0}$ ($4.53\cdot10^{-1}$)	$9.80\cdot10^{-1}$ ($5.00\cdot10^{-1}$)	$8.84\cdot10^{-4}$	$2.91\cdot10^{-6}$	$8.16\cdot10^{-2}$
95% CI	($1.40\cdot10^{0}$-$1.75\cdot10^{0}$)	($1.04\cdot10^{0}$-$1.43\cdot10^{0}$)	($7.64\cdot10^{-1}$-$1.20\cdot10^{0}$)			
Triglycerides, LDL-1						
Mean (SD)	$4.91\cdot10^{0}$ ($8.08\cdot10^{-1}$)	$4.59\cdot10^{0}$ ($8.36\cdot10^{-1}$)	$4.79\cdot10^{0}$ ($8.66\cdot10^{-1}$)	$5.04\cdot10^{-2}$	$4.90\cdot10^{-1}$	$3.88\cdot10^{-1}$
95% CI	($4.56\cdot10^{0}$-$5.26\cdot10^{0}$)	($4.22\cdot10^{0}$-$4.95\cdot10^{0}$)	($4.42\cdot10^{0}$-$5.16\cdot10^{0}$)			
Triglycerides, LDL-2						
Mean (SD)	$2.02\cdot10^{0}$ ($5.06\cdot10^{-1}$)	$2.07\cdot10^{0}$ ($5.52\cdot10^{-1}$)	$2.19\cdot10^{0}$ ($6.16\cdot10^{-1}$)	$6.43\cdot10^{-1}$	$7.51\cdot10^{-2}$	$2.94\cdot10^{-1}$
95% CI	($1.80\cdot10^{0}$-$2.23\cdot10^{0}$)	($1.83\cdot10^{0}$-$2.30\cdot10^{0}$)	($1.93\cdot10^{0}$-$2.46\cdot10^{0}$)			
Triglycerides, LDL-3						
Mean (SD)	$2.19\cdot10^{0}$ ($3.76\cdot10^{-1}$)	$2.02\cdot10^{0}$ ($4.09\cdot10^{-1}$)	$1.98\cdot10^{0}$ ($4.20\cdot10^{-1}$)	$6.57\cdot10^{-2}$	$2.14\cdot10^{-2}$	$9.93\cdot10^{-1}$
95% CI	($2.02\cdot10^{0}$-$2.35\cdot10^{0}$)	($1.84\cdot10^{0}$-$2.20\cdot10^{0}$)	($1.80\cdot10^{0}$-$2.16\cdot10^{0}$)			

Triglycerides, LDL-4						
Mean (SD)<br>95% CI	$1.74\cdot10^{0}$ ($5.34\cdot10^{-1}$)<br>($1.51\cdot10^{0}$-$1.97\cdot10^{0}$)	$1.80\cdot10^{0}$ ($5.56\cdot10^{-1}$)<br>($1.56\cdot10^{0}$-$2.04\cdot10^{0}$)	$1.64\cdot10^{0}$ ($6.20\cdot10^{-1}$)<br>($1.37\cdot10^{0}$-$1.91\cdot10^{0}$)	$6.43\cdot10^{-1}$	$4.50\cdot10^{-1}$	$3.01\cdot10^{-1}$
Triglycerides, LDL-5						
Mean (SD)<br>95% CI	$1.65\cdot10^{0}$ ($7.26\cdot10^{-1}$)<br>($1.33\cdot10^{0}$-$1.96\cdot10^{0}$)	$1.80\cdot10^{0}$ ($7.67\cdot10^{-1}$)<br>($1.46\cdot10^{0}$-$2.13\cdot10^{0}$)	$1.74\cdot10^{0}$ ($8.42\cdot10^{-1}$)<br>($1.38\cdot10^{0}$-$2.10\cdot10^{0}$)	$1.58\cdot10^{-1}$	$4.80\cdot10^{-1}$	$9.93\cdot10^{-1}$
Triglycerides, LDL-6						
Mean (SD)<br>95% CI	$2.63\cdot10^{0}$ ($7.48\cdot10^{-1}$)<br>($2.31\cdot10^{0}$-$2.95\cdot10^{0}$)	$2.80\cdot10^{0}$ ($8.42\cdot10^{-1}$)<br>($2.44\cdot10^{0}$-$3.16\cdot10^{0}$)	$2.80\cdot10^{0}$ ($9.14\cdot10^{-1}$)<br>($2.41\cdot10^{0}$-$3.20\cdot10^{0}$)	$2.28\cdot10^{-1}$	$2.45\cdot10^{-1}$	$9.95\cdot10^{-1}$
Cholesterol, LDL-1						
Mean (SD)<br>95% CI	$2.16\cdot10^{1}$ ($4.25\cdot10^{0}$)<br>($1.97\cdot10^{1}$-$2.34\cdot10^{1}$)	$2.18\cdot10^{1}$ ($4.46\cdot10^{0}$)<br>($1.98\cdot10^{1}$-$2.37\cdot10^{1}$)	$2.29\cdot10^{1}$ ($5.22\cdot10^{0}$)<br>($2.07\cdot10^{1}$-$2.52\cdot10^{1}$)	$8.51\cdot10^{-1}$	$1.77\cdot10^{-1}$	$3.88\cdot10^{-1}$
Cholesterol, LDL-2						
Mean (SD)<br>95% CI	$1.43\cdot10^{1}$ ($4.89\cdot10^{0}$)<br>($1.22\cdot10^{1}$-$1.64\cdot10^{1}$)	$1.49\cdot10^{1}$ ($5.16\cdot10^{0}$)<br>($1.27\cdot10^{1}$-$1.71\cdot10^{1}$)	$1.69\cdot10^{1}$ ($6.16\cdot10^{0}$)<br>($1.43\cdot10^{1}$-$1.96\cdot10^{1}$)	$5.58\cdot10^{-1}$	$6.06\cdot10^{-3}$	$8.16\cdot10^{-2}$
Cholesterol, LDL-3						
Mean (SD)<br>95% CI	$1.57\cdot10^{1}$ ($5.39\cdot10^{0}$)<br>($1.34\cdot10^{1}$-$1.81\cdot10^{1}$)	$1.74\cdot10^{1}$ ($5.85\cdot10^{0}$)<br>($1.48\cdot10^{1}$-$1.99\cdot10^{1}$)	$1.70\cdot10^{1}$ ($6.48\cdot10^{0}$)<br>($1.42\cdot10^{1}$-$1.98\cdot10^{1}$)	$9.37\cdot10^{-2}$	$2.47\cdot10^{-1}$	$9.93\cdot10^{-1}$
Cholesterol, LDL-4						
Mean (SD)<br>95% CI	$1.29\cdot10^{1}$ ($6.61\cdot10^{0}$)<br>($1.01\cdot10^{1}$-$1.58\cdot10^{1}$)	$1.51\cdot10^{1}$ ($7.11\cdot10^{0}$)<br>($1.20\cdot10^{1}$-$1.82\cdot10^{1}$)	$1.29\cdot10^{1}$ ($7.25\cdot10^{0}$)<br>($9.80\cdot10^{0}$-$1.61\cdot10^{1}$)	$3.91\cdot10^{-2}$	$9.97\cdot10^{-1}$	$1.72\cdot10^{-1}$
Cholesterol, LDL-5						
Mean (SD)<br>95% CI	$9.18\cdot10^{0}$ ($6.20\cdot10^{0}$)<br>($6.50\cdot10^{0}$-$1.19\cdot10^{1}$)	$1.15\cdot10^{1}$ ($6.62\cdot10^{0}$)<br>($8.60\cdot10^{0}$-$1.43\cdot10^{1}$)	$1.06\cdot10^{1}$ ($6.62\cdot10^{0}$)<br>($7.76\cdot10^{0}$-$1.35\cdot10^{1}$)	$1.03\cdot10^{-3}$	$7.74\cdot10^{-2}$	$5.13\cdot10^{-1}$
Cholesterol, LDL-6						
Mean (SD)<br>95% CI	$1.64\cdot10^{1}$ ($5.91\cdot10^{0}$)<br>($1.38\cdot10^{1}$-$1.89\cdot10^{1}$)	$1.82\cdot10^{1}$ ($6.44\cdot10^{0}$)<br>($1.55\cdot10^{1}$-$2.10\cdot10^{1}$)	$1.82\cdot10^{1}$ ($6.32\cdot10^{0}$)<br>($1.55\cdot10^{1}$-$2.10\cdot10^{1}$)	$1.41\cdot10^{-2}$	$1.88\cdot10^{-2}$	$9.95\cdot10^{-1}$
Free Cholesterol, LDL-1						
Mean (SD)<br>95% CI	$6.42\cdot10^{0}$ ($1.26\cdot10^{0}$)<br>($5.87\cdot10^{0}$-$6.96\cdot10^{0}$)	$6.78\cdot10^{0}$ ($1.35\cdot10^{0}$)<br>($6.20\cdot10^{0}$-$7.37\cdot10^{0}$)	$7.31\cdot10^{0}$ ($1.58\cdot10^{0}$)<br>($6.63\cdot10^{0}$-$7.99\cdot10^{0}$)	$1.71\cdot10^{-1}$	$3.94\cdot10^{-3}$	$1.69\cdot10^{-1}$
Free Cholesterol, LDL-2						
Mean (SD)<br>95% CI	$4.41\cdot10^{0}$ ($1.64\cdot10^{0}$)<br>($3.70\cdot10^{0}$-$5.12\cdot10^{0}$)	$4.87\cdot10^{0}$ ($1.72\cdot10^{0}$)<br>($4.13\cdot10^{0}$-$5.62\cdot10^{0}$)	$5.73\cdot10^{0}$ ($1.99\cdot10^{0}$)<br>($4.87\cdot10^{0}$-$6.59\cdot10^{0}$)	$9.37\cdot10^{-2}$	$6.87\cdot10^{-5}$	$4.08\cdot10^{-2}$
Free Cholesterol, LDL-3						
Mean (SD)<br>95% CI	$5.29\cdot10^{0}$ ($1.42\cdot10^{0}$)<br>($4.68\cdot10^{0}$-$5.91\cdot10^{0}$)	$5.73\cdot10^{0}$ ($1.55\cdot10^{0}$)<br>($5.06\cdot10^{0}$-$6.40\cdot10^{0}$)	$5.86\cdot10^{0}$ ($1.70\cdot10^{0}$)<br>($5.12\cdot10^{0}$-$6.59\cdot10^{0}$)	$9.37\cdot10^{-2}$	$6.57\cdot10^{-2}$	$9.93\cdot10^{-1}$
Free Cholesterol, LDL-4						
Mean (SD)<br>95% CI	$3.98\cdot10^{0}$ ($1.44\cdot10^{0}$)<br>($3.35\cdot10^{0}$-$4.60\cdot10^{0}$)	$4.66\cdot10^{0}$ ($1.55\cdot10^{0}$)<br>($3.98\cdot10^{0}$-$5.33\cdot10^{0}$)	$4.27\cdot10^{0}$ ($1.60\cdot10^{0}$)<br>($3.58\cdot10^{0}$-$4.97\cdot10^{0}$)	$1.05\cdot10^{-2}$	$3.55\cdot10^{-1}$	$3.88\cdot10^{-1}$
Free Cholesterol, LDL-5						
Mean (SD)<br>95% CI	$2.89\cdot10^{0}$ ($1.38\cdot10^{0}$)<br>($2.29\cdot10^{0}$-$3.49\cdot10^{0}$)	$3.51\cdot10^{0}$ ($1.46\cdot10^{0}$)<br>($2.88\cdot10^{0}$-$4.14\cdot10^{0}$)	$3.46\cdot10^{0}$ ($1.44\cdot10^{0}$)<br>($2.83\cdot10^{0}$-$4.08\cdot10^{0}$)	$1.33\cdot10^{-3}$	$9.61\cdot10^{-3}$	$9.95\cdot10^{-1}$
Free Cholesterol, LDL-6						
Mean (SD)<br>95% CI	$4.10\cdot10^{0}$ ($1.35\cdot10^{0}$)<br>($3.52\cdot10^{0}$-$4.69\cdot10^{0}$)	$4.81\cdot10^{0}$ ($1.46\cdot10^{0}$)<br>($4.18\cdot10^{0}$-$5.44\cdot10^{0}$)	$5.06\cdot10^{0}$ ($1.40\cdot10^{0}$)<br>($4.45\cdot10^{0}$-$5.67\cdot10^{0}$)	$1.46\cdot10^{-3}$	$2.32\cdot10^{-5}$	$4.21\cdot10^{-1}$
Phospholipids, LDL-1						
Mean (SD)<br>95% CI	$1.25\cdot10^{1}$ ($2.12\cdot10^{0}$)<br>($1.15\cdot10^{1}$-$1.34\cdot10^{1}$)	$1.24\cdot10^{1}$ ($2.24\cdot10^{0}$)<br>($1.14\cdot10^{1}$-$1.34\cdot10^{1}$)	$1.31\cdot10^{1}$ ($2.59\cdot10^{0}$)<br>($1.19\cdot10^{1}$-$1.42\cdot10^{1}$)	$8.97\cdot10^{-1}$	$2.38\cdot10^{-1}$	$3.05\cdot10^{-1}$
Phospholipids, LDL-2						
Mean (SD)<br>95% CI	$8.44\cdot10^{0}$ ($2.51\cdot10^{0}$)<br>($7.36\cdot10^{0}$-$9.53\cdot10^{0}$)	$8.69\cdot10^{0}$ ($2.66\cdot10^{0}$)<br>($7.54\cdot10^{0}$-$9.85\cdot10^{0}$)	$9.73\cdot10^{0}$ ($3.13\cdot10^{0}$)<br>($8.38\cdot10^{0}$-$1.11\cdot10^{1}$)	$6.38\cdot10^{-1}$	$8.08\cdot10^{-3}$	$8.16\cdot10^{-2}$
Phospholipids, LDL-3						
Mean (SD)<br>95% CI	$9.02\cdot10^{0}$ ($2.63\cdot10^{0}$)<br>($7.88\cdot10^{0}$-$1.02\cdot10^{1}$)	$9.72\cdot10^{0}$ ($2.84\cdot10^{0}$)<br>($8.49\cdot10^{0}$-$1.09\cdot10^{1}$)	$9.59\cdot10^{0}$ ($3.16\cdot10^{0}$)<br>($8.22\cdot10^{0}$-$1.10\cdot10^{1}$)	$1.49\cdot10^{-1}$	$3.03\cdot10^{-1}$	$9.95\cdot10^{-1}$
Phospholipids, LDL-4						

Mean (SD)<br>95% CI	$7.63\cdot10^{0}$ ($3.34\cdot10^{0}$)<br>($6.19\cdot10^{0}$-$9.08\cdot10^{0}$)	$8.63\cdot10^{0}$ ($3.58\cdot10^{0}$)<br>($7.08\cdot10^{0}$-$1.02\cdot10^{1}$)	$7.49\cdot10^{0}$ ($3.66\cdot10^{0}$)<br>($5.91\cdot10^{0}$-$9.08\cdot10^{0}$)	$5.31\cdot10^{-2}$	$8.25\cdot10^{-1}$	$1.69\cdot10^{-1}$
Phospholipids, LDL-5						
Mean (SD)<br>95% CI	$5.47\cdot10^{0}$ ($3.02\cdot10^{0}$)<br>($4.16\cdot10^{0}$-$6.77\cdot10^{0}$)	$6.53\cdot10^{0}$ ($3.23\cdot10^{0}$)<br>($5.13\cdot10^{0}$-$7.92\cdot10^{0}$)	$6.15\cdot10^{0}$ ($3.25\cdot10^{0}$)<br>($4.75\cdot10^{0}$-$7.56\cdot10^{0}$)	$1.93\cdot10^{-3}$	$9.02\cdot10^{-2}$	$6.23\cdot10^{-1}$
Phospholipids, LDL-6						
Mean (SD)<br>95% CI	$9.64\cdot10^{0}$ ($2.63\cdot10^{0}$)<br>($8.50\cdot10^{0}$-$1.08\cdot10^{1}$)	$1.06\cdot10^{1}$ ($2.89\cdot10^{0}$)<br>($9.35\cdot10^{0}$-$1.18\cdot10^{1}$)	$1.08\cdot10^{1}$ ($2.88\cdot10^{0}$)<br>($9.53\cdot10^{0}$-$1.20\cdot10^{1}$)	$1.33\cdot10^{-2}$	$5.82\cdot10^{-3}$	$9.93\cdot10^{-1}$
Apo-B, LDL-1						
Mean (SD)<br>95% CI	$1.13\cdot10^{1}$ ($2.09\cdot10^{0}$)<br>($1.04\cdot10^{1}$-$1.23\cdot10^{1}$)	$1.13\cdot10^{1}$ ($2.21\cdot10^{0}$)<br>($1.03\cdot10^{1}$-$1.22\cdot10^{1}$)	$1.20\cdot10^{1}$ ($2.51\cdot10^{0}$)<br>($1.09\cdot10^{1}$-$1.30\cdot10^{1}$)	$8.70\cdot10^{-1}$	$2.00\cdot10^{-1}$	$2.50\cdot10^{-1}$
Apo-B, LDL-2						
Mean (SD)<br>95% CI	$7.97\cdot10^{0}$ ($2.41\cdot10^{0}$)<br>($6.93\cdot10^{0}$-$9.01\cdot10^{0}$)	$8.14\cdot10^{0}$ ($2.51\cdot10^{0}$)<br>($7.06\cdot10^{0}$-$9.22\cdot10^{0}$)	$9.20\cdot10^{0}$ ($3.01\cdot10^{0}$)<br>($7.90\cdot10^{0}$-$1.05\cdot10^{1}$)	$7.13\cdot10^{-1}$	$9.61\cdot10^{-3}$	$8.16\cdot10^{-2}$
Apo-B, LDL-3						
Mean (SD)<br>95% CI	$8.96\cdot10^{0}$ ($2.80\cdot10^{0}$)<br>($7.75\cdot10^{0}$-$1.02\cdot10^{1}$)	$9.73\cdot10^{0}$ ($3.06\cdot10^{0}$)<br>($8.41\cdot10^{0}$-$1.11\cdot10^{1}$)	$9.66\cdot10^{0}$ ($3.38\cdot10^{0}$)<br>($8.20\cdot10^{0}$-$1.11\cdot10^{1}$)	$1.35\cdot10^{-1}$	$2.31\cdot10^{-1}$	$9.95\cdot10^{-1}$
Apo-B, LDL-4						
Mean (SD)<br>95% CI	$8.14\cdot10^{0}$ ($3.98\cdot10^{0}$)<br>($6.42\cdot10^{0}$-$9.86\cdot10^{0}$)	$9.46\cdot10^{0}$ ($4.27\cdot10^{0}$)<br>($7.61\cdot10^{0}$-$1.13\cdot10^{1}$)	$8.35\cdot10^{0}$ ($4.39\cdot10^{0}$)<br>($6.45\cdot10^{0}$-$1.02\cdot10^{1}$)	$4.07\cdot10^{-2}$	$8.00\cdot10^{-1}$	$2.50\cdot10^{-1}$
Apo-B, LDL-5						
Mean (SD)<br>95% CI	$6.35\cdot10^{0}$ ($4.13\cdot10^{0}$)<br>($4.56\cdot10^{0}$-$8.13\cdot10^{0}$)	$7.82\cdot10^{0}$ ($4.38\cdot10^{0}$)<br>($5.93\cdot10^{0}$-$9.71\cdot10^{0}$)	$7.52\cdot10^{0}$ ($4.43\cdot10^{0}$)<br>($5.60\cdot10^{0}$-$9.44\cdot10^{0}$)	$1.33\cdot10^{-3}$	$2.85\cdot10^{-2}$	$9.36\cdot10^{-1}$
Apo-B, LDL-6						
Mean (SD)<br>95% CI	$1.35\cdot10^{1}$ ($4.68\cdot10^{0}$)<br>($1.15\cdot10^{1}$-$1.55\cdot10^{1}$)	$1.47\cdot10^{1}$ ($5.04\cdot10^{0}$)<br>($1.25\cdot10^{1}$-$1.68\cdot10^{1}$)	$1.47\cdot10^{1}$ ($4.97\cdot10^{0}$)<br>($1.26\cdot10^{1}$-$1.69\cdot10^{1}$)	$5.23\cdot10^{-2}$	$4.93\cdot10^{-2}$	$9.95\cdot10^{-1}$
Triglycerides, HDL-1						
Mean (SD)<br>95% CI	$2.04\cdot10^{0}$ ($1.09\cdot10^{0}$)<br>($1.57\cdot10^{0}$-$2.51\cdot10^{0}$)	$2.26\cdot10^{0}$ ($1.20\cdot10^{0}$)<br>($1.75\cdot10^{0}$-$2.78\cdot10^{0}$)	$2.34\cdot10^{0}$ ($1.28\cdot10^{0}$)<br>($1.79\cdot10^{0}$-$2.89\cdot10^{0}$)	$2.44\cdot10^{-1}$	$1.36\cdot10^{-1}$	$9.93\cdot10^{-1}$
Triglycerides, HDL-2						
Mean (SD)<br>95% CI	$9.81\cdot10^{-1}$ ($3.19\cdot10^{-1}$)<br>($8.43\cdot10^{-1}$-$1.12\cdot10^{0}$)	$1.09\cdot10^{0}$ ($3.47\cdot10^{-1}$)<br>($9.41\cdot10^{-1}$-$1.24\cdot10^{0}$)	$1.12\cdot10^{0}$ ($3.79\cdot10^{-1}$)<br>($9.59\cdot10^{-1}$-$1.29\cdot10^{0}$)	$1.65\cdot10^{-1}$	$1.04\cdot10^{-1}$	$9.93\cdot10^{-1}$
Triglycerides, HDL-3						
Mean (SD)<br>95% CI	$1.49\cdot10^{0}$ ($2.42\cdot10^{-1}$)<br>($1.38\cdot10^{0}$-$1.59\cdot10^{0}$)	$1.53\cdot10^{0}$ ($2.77\cdot10^{-1}$)<br>($1.41\cdot10^{0}$-$1.65\cdot10^{0}$)	$1.55\cdot10^{0}$ ($3.15\cdot10^{-1}$)<br>($1.42\cdot10^{0}$-$1.69\cdot10^{0}$)	$6.54\cdot10^{-1}$	$4.44\cdot10^{-1}$	$9.93\cdot10^{-1}$
Triglycerides, HDL-4						
Mean (SD)<br>95% CI	$2.83\cdot10^{0}$ ($7.23\cdot10^{-1}$)<br>($2.52\cdot10^{0}$-$3.14\cdot10^{0}$)	$2.52\cdot10^{0}$ ($8.13\cdot10^{-1}$)<br>($2.17\cdot10^{0}$-$2.88\cdot10^{0}$)	$2.45\cdot10^{0}$ ($8.72\cdot10^{-1}$)<br>($2.07\cdot10^{0}$-$2.83\cdot10^{0}$)	$8.39\cdot10^{-3}$	$1.57\cdot10^{-3}$	$9.09\cdot10^{-1}$
Cholesterol, HDL-1						
Mean (SD)<br>95% CI	$1.71\cdot10^{1}$ ($7.20\cdot10^{0}$)<br>($1.40\cdot10^{1}$-$2.02\cdot10^{1}$)	$1.95\cdot10^{1}$ ($8.15\cdot10^{0}$)<br>($1.60\cdot10^{1}$-$2.31\cdot10^{1}$)	$2.12\cdot10^{1}$ ($8.97\cdot10^{0}$)<br>($1.73\cdot10^{1}$-$2.51\cdot10^{1}$)	$6.93\cdot10^{-6}$	$1.71\cdot10^{-7}$	$3.01\cdot10^{-2}$
Cholesterol, HDL-2						
Mean (SD)<br>95% CI	$7.80\cdot10^{0}$ ($2.47\cdot10^{0}$)<br>($6.73\cdot10^{0}$-$8.87\cdot10^{0}$)	$8.82\cdot10^{0}$ ($2.80\cdot10^{0}$)<br>($7.61\cdot10^{0}$-$1.00\cdot10^{1}$)	$9.57\cdot10^{0}$ ($3.10\cdot10^{0}$)<br>($8.23\cdot10^{0}$-$1.09\cdot10^{1}$)	$3.66\cdot10^{-4}$	$1.79\cdot10^{-6}$	$7.04\cdot10^{-2}$
Cholesterol, HDL-3						
Mean (SD)<br>95% CI	$1.10\cdot10^{1}$ ($1.85\cdot10^{0}$)<br>($1.02\cdot10^{1}$-$1.18\cdot10^{1}$)	$1.20\cdot10^{1}$ ($2.09\cdot10^{0}$)<br>($1.11\cdot10^{1}$-$1.29\cdot10^{1}$)	$1.26\cdot10^{1}$ ($2.42\cdot10^{0}$)<br>($1.15\cdot10^{1}$-$1.36\cdot10^{1}$)	$2.46\cdot10^{-3}$	$1.16\cdot10^{-4}$	$2.35\cdot10^{-1}$
Cholesterol, HDL-4						
Mean (SD)<br>95% CI	$2.20\cdot10^{1}$ ($2.78\cdot10^{0}$)<br>($2.08\cdot10^{1}$-$2.32\cdot10^{1}$)	$2.22\cdot10^{1}$ ($2.83\cdot10^{0}$)<br>($2.09\cdot10^{1}$-$2.34\cdot10^{1}$)	$2.23\cdot10^{1}$ ($3.21\cdot10^{0}$)<br>($2.09\cdot10^{1}$-$2.37\cdot10^{1}$)	$7.82\cdot10^{-1}$	$5.69\cdot10^{-1}$	$9.93\cdot10^{-1}$
Free Cholesterol, HDL-1						
Mean (SD)<br>95% CI	$4.21\cdot10^{0}$ ($1.59\cdot10^{0}$)<br>($3.53\cdot10^{0}$-$4.90\cdot10^{0}$)	$5.10\cdot10^{0}$ ($1.84\cdot10^{0}$)<br>($4.30\cdot10^{0}$-$5.89\cdot10^{0}$)	$5.63\cdot10^{0}$ ($2.08\cdot10^{0}$)<br>($4.73\cdot10^{0}$-$6.53\cdot10^{0}$)	$6.93\cdot10^{-6}$	$1.71\cdot10^{-7}$	$4.93\cdot10^{-2}$
Free Cholesterol, HDL-2						
Mean (SD)<br>95% CI	$1.96\cdot10^{0}$ ($5.87\cdot10^{-1}$)<br>($1.71\cdot10^{0}$-$2.22\cdot10^{0}$)	$2.39\cdot10^{0}$ ($6.79\cdot10^{-1}$)<br>($2.10\cdot10^{0}$-$2.69\cdot10^{0}$)	$2.67\cdot10^{0}$ ($7.76\cdot10^{-1}$)<br>($2.33\cdot10^{0}$-$3.00\cdot10^{0}$)	$7.58\cdot10^{-5}$	$2.50\cdot10^{-7}$	$7.63\cdot10^{-2}$

Free Cholesterol, HDL-3						
Mean (SD)	$2.23\cdot10^{0}$ ($4.66\cdot10^{-1}$)	$2.59\cdot10^{0}$ ($5.12\cdot10^{-1}$)	$2.79\cdot10^{0}$ ($6.11\cdot10^{-1}$)	$4.34\cdot10^{-4}$	$5.12\cdot10^{-6}$	$1.69\cdot10^{-1}$
95% CI	($2.03\cdot10^{0}$-$2.44\cdot10^{0}$)	($2.37\cdot10^{0}$-$2.81\cdot10^{0}$)	($2.53\cdot10^{0}$-$3.06\cdot10^{0}$)			
Free Cholesterol, HDL-4						
Mean (SD)	$4.00\cdot10^{0}$ ($8.05\cdot10^{-1}$)	$4.16\cdot10^{0}$ ($8.49\cdot10^{-1}$)	$4.23\cdot10^{0}$ ($8.93\cdot10^{-1}$)	$1.58\cdot10^{-1}$	$6.08\cdot10^{-2}$	$9.93\cdot10^{-1}$
95% CI	($3.66\cdot10^{0}$-$4.35\cdot10^{0}$)	($3.79\cdot10^{0}$-$4.53\cdot10^{0}$)	($3.84\cdot10^{0}$-$4.61\cdot10^{0}$)			
Phospholipids, HDL-1						
Mean (SD)	$2.14\cdot10^{1}$ ($9.37\cdot10^{0}$)	$2.40\cdot10^{1}$ ($1.05\cdot10^{1}$)	$2.61\cdot10^{1}$ ($1.14\cdot10^{1}$)	$7.69\cdot10^{-5}$	$5.14\cdot10^{-7}$	$3.01\cdot10^{-2}$
95% CI	($1.73\cdot10^{1}$-$2.54\cdot10^{1}$)	($1.95\cdot10^{1}$-$2.86\cdot10^{1}$)	($2.12\cdot10^{1}$-$3.10\cdot10^{1}$)			
Phospholipids, HDL-2						
Mean (SD)	$1.28\cdot10^{1}$ ($3.82\cdot10^{0}$)	$1.40\cdot10^{1}$ ($4.26\cdot10^{0}$)	$1.50\cdot10^{1}$ ($4.69\cdot10^{0}$)	$2.09\cdot10^{-3}$	$1.96\cdot10^{-5}$	$8.16\cdot10^{-2}$
95% CI	($1.11\cdot10^{1}$-$1.44\cdot10^{1}$)	($1.21\cdot10^{1}$-$1.58\cdot10^{1}$)	($1.30\cdot10^{1}$-$1.70\cdot10^{1}$)			
Phospholipids, HDL-3						
Mean (SD)	$1.79\cdot10^{1}$ ($3.10\cdot10^{0}$)	$1.91\cdot10^{1}$ ($3.42\cdot10^{0}$)	$2.01\cdot10^{1}$ ($3.95\cdot10^{0}$)	$2.41\cdot10^{-2}$	$5.05\cdot10^{-4}$	$1.98\cdot10^{-1}$
95% CI	($1.66\cdot10^{1}$-$1.93\cdot10^{1}$)	($1.76\cdot10^{1}$-$2.06\cdot10^{1}$)	($1.84\cdot10^{1}$-$2.18\cdot10^{1}$)			
Phospholipids, HDL-4						
Mean (SD)	$3.11\cdot10^{1}$ ($3.24\cdot10^{0}$)	$3.08\cdot10^{1}$ ($3.31\cdot10^{0}$)	$3.12\cdot10^{1}$ ($3.84\cdot10^{0}$)	$6.83\cdot10^{-1}$	$8.50\cdot10^{-1}$	$9.36\cdot10^{-1}$
95% CI	($2.97\cdot10^{1}$-$3.25\cdot10^{1}$)	($2.94\cdot10^{1}$-$3.23\cdot10^{1}$)	($2.95\cdot10^{1}$-$3.28\cdot10^{1}$)			
Apo-A1, HDL-1						
Mean (SD)	$2.45\cdot10^{1}$ ($1.35\cdot10^{1}$)	$2.85\cdot10^{1}$ ($1.50\cdot10^{1}$)	$3.19\cdot10^{1}$ ($1.65\cdot10^{1}$)	$7.58\cdot10^{-5}$	$1.71\cdot10^{-7}$	$1.72\cdot10^{-2}$
95% CI	($1.87\cdot10^{1}$-$3.03\cdot10^{1}$)	($2.20\cdot10^{1}$-$3.50\cdot10^{1}$)	($2.48\cdot10^{1}$-$3.90\cdot10^{1}$)			
Apo-A1, HDL-2						
Mean (SD)	$1.99\cdot10^{1}$ ($4.14\cdot10^{0}$)	$2.10\cdot10^{1}$ ($4.62\cdot10^{0}$)	$2.20\cdot10^{1}$ ($5.20\cdot10^{0}$)	$2.89\cdot10^{-2}$	$1.12\cdot10^{-3}$	$2.27\cdot10^{-1}$
95% CI	($1.81\cdot10^{1}$-$2.16\cdot10^{1}$)	($1.90\cdot10^{1}$-$2.30\cdot10^{1}$)	($1.98\cdot10^{1}$-$2.43\cdot10^{1}$)			
Apo-A1, HDL-3						
Mean (SD)	$2.87\cdot10^{1}$ ($4.38\cdot10^{0}$)	$3.03\cdot10^{1}$ ($4.82\cdot10^{0}$)	$3.10\cdot10^{1}$ ($5.50\cdot10^{0}$)	$4.07\cdot10^{-2}$	$8.91\cdot10^{-3}$	$6.26\cdot10^{-1}$
95% CI	($2.68\cdot10^{1}$-$3.06\cdot10^{1}$)	($2.82\cdot10^{1}$-$3.23\cdot10^{1}$)	($2.87\cdot10^{1}$-$3.34\cdot10^{1}$)			
Apo-A1, HDL-4						
Mean (SD)	$7.88\cdot10^{1}$ ($9.14\cdot10^{0}$)	$7.94\cdot10^{1}$ ($9.35\cdot10^{0}$)	$7.92\cdot10^{1}$ ($1.08\cdot10^{1}$)	$6.83\cdot10^{-1}$	$8.25\cdot10^{-1}$	$9.95\cdot10^{-1}$
95% CI	($7.49\cdot10^{1}$-$8.28\cdot10^{1}$)	($7.54\cdot10^{1}$-$8.35\cdot10^{1}$)	($7.46\cdot10^{1}$-$8.39\cdot10^{1}$)			
Apo-A2, HDL-1						
Mean (SD)	$2.53\cdot10^{0}$ ($1.30\cdot10^{0}$)	$3.02\cdot10^{0}$ ($1.47\cdot10^{0}$)	$3.32\cdot10^{0}$ ($1.62\cdot10^{0}$)	$3.66\cdot10^{-4}$	$5.31\cdot10^{-6}$	$1.28\cdot10^{-1}$
95% CI	($1.96\cdot10^{0}$-$3.09\cdot10^{0}$)	($2.39\cdot10^{0}$-$3.66\cdot10^{0}$)	($2.62\cdot10^{0}$-$4.01\cdot10^{0}$)			
Apo-A2, HDL-2						
Mean (SD)	$3.31\cdot10^{0}$ ($9.09\cdot10^{-1}$)	$3.90\cdot10^{0}$ ($1.04\cdot10^{0}$)	$4.22\cdot10^{0}$ ($1.22\cdot10^{0}$)	$4.34\cdot10^{-4}$	$1.22\cdot10^{-5}$	$1.69\cdot10^{-1}$
95% CI	($2.91\cdot10^{0}$-$3.70\cdot10^{0}$)	($3.45\cdot10^{0}$-$4.34\cdot10^{0}$)	($3.70\cdot10^{0}$-$4.75\cdot10^{0}$)			
Apo-A2, HDL-3						
Mean (SD)	$6.27\cdot10^{0}$ ($1.08\cdot10^{0}$)	$7.08\cdot10^{0}$ ($1.20\cdot10^{0}$)	$7.52\cdot10^{0}$ ($1.48\cdot10^{0}$)	$4.44\cdot10^{-4}$	$2.10\cdot10^{-5}$	$2.01\cdot10^{-1}$
95% CI	($5.81\cdot10^{0}$-$6.74\cdot10^{0}$)	($6.57\cdot10^{0}$-$7.60\cdot10^{0}$)	($6.88\cdot10^{0}$-$8.16\cdot10^{0}$)			
Apo-A2, HDL-4						
Mean (SD)	$2.02\cdot10^{1}$ ($3.30\cdot10^{0}$)	$2.07\cdot10^{1}$ ($3.35\cdot10^{0}$)	$2.11\cdot10^{1}$ ($3.85\cdot10^{0}$)	$1.57\cdot10^{-1}$	$5.65\cdot10^{-2}$	$6.23\cdot10^{-1}$
95% CI	($1.88\cdot10^{1}$-$2.16\cdot10^{1}$)	($1.92\cdot10^{1}$-$2.21\cdot10^{1}$)	($1.94\cdot10^{1}$-$2.28\cdot10^{1}$)			

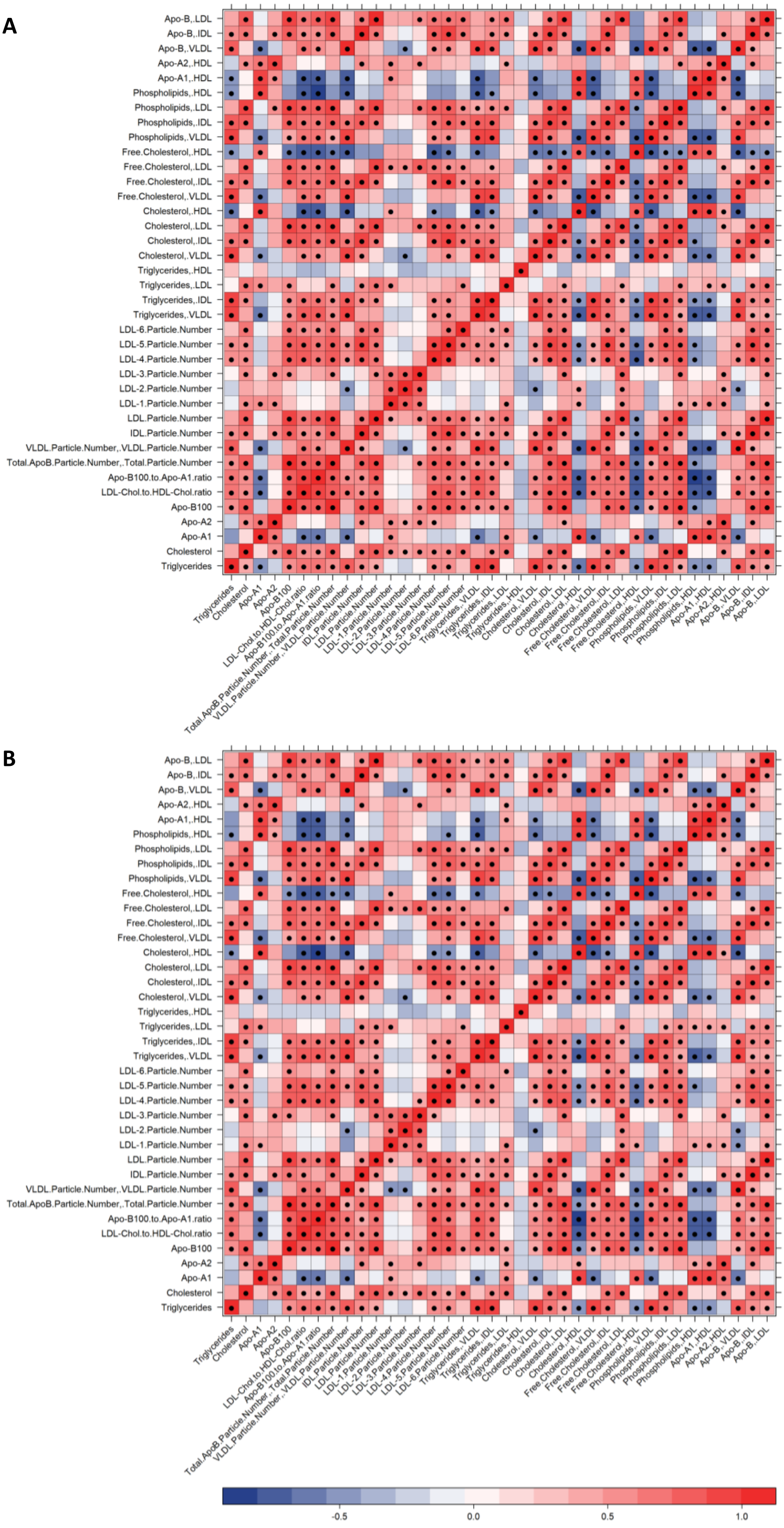
A
B
Apo-B,.LDL
Apo-B,.IDL
Apo-B,.VLDL
Apo-A2,.HDL
Apo-A1,.HDL
Phospholipids,.HDL
Phospholipids,.LDL
Phospholipids,.IDL
Phospholipids,.VLDL
Free.Cholesterol,.HDL
Free.Cholesterol,.LDL
Free.Cholesterol,.IDL
Free.Cholesterol,.VLDL
Cholesterol,.HDL
Cholesterol,.LDL
Cholesterol,.IDL
Cholesterol,.VLDL
Triglycerides,.HDL
Triglycerides,.LDL
Triglycerides,.IDL
Triglycerides,.VLDL
LDL-6.Particle.Number
LDL-5.Particle.Number
LDL-4.Particle.Number
LDL-3.Particle.Number
LDL-2.Particle.Number
LDL-1.Particle.Number
LDL.Particle.Number
IDL.Particle.Number
VLDL.Particle.Number,.VLDL.Particle.Number
Total.ApoB.Particle.Number,.Total.Particle.Number
Apo-B100.to.Apo-A1.ratio
LDL-Chol.to.HDL-Chol.ratio
Apo-B100
Apo-A2
Apo-A1
Cholesterol
Triglycerides
-0.5
0.0
0.5
1.0

C

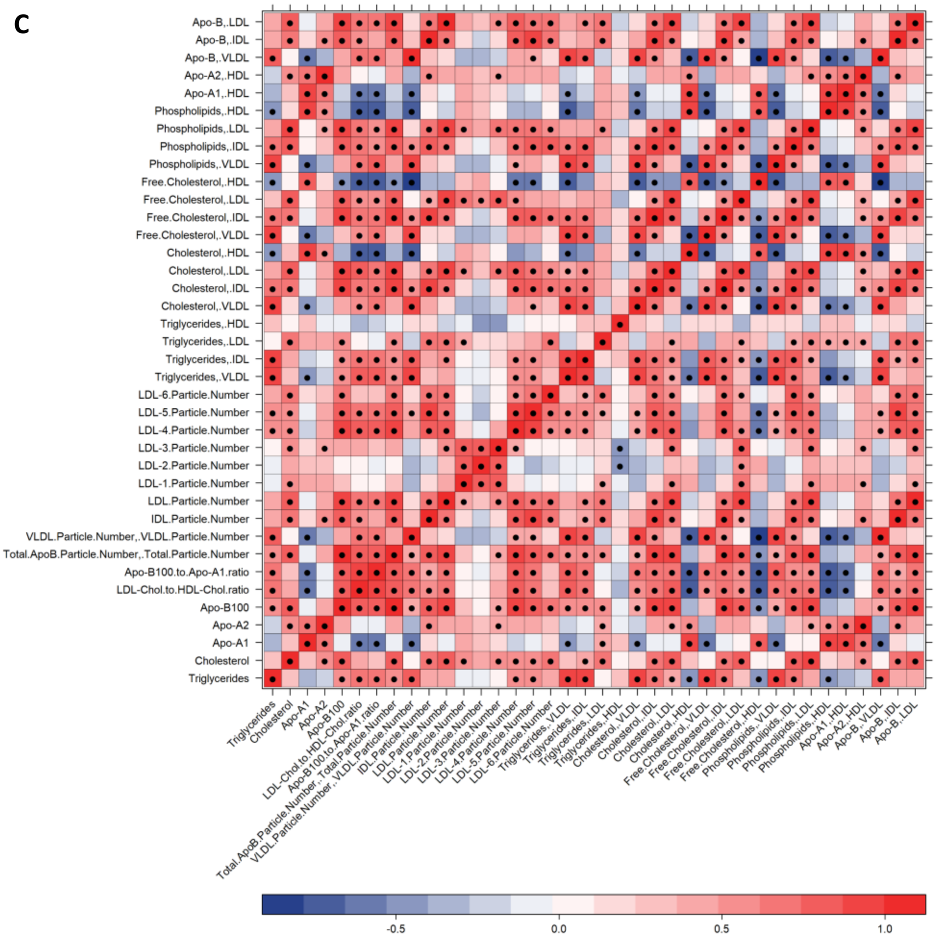

Figure S1. Heatmap showing correlations among lipoprotein-related parameters in the three blood derivatives: A) citrate plasma, B) EDTA plasma, C) serum. R values are shown as different degree of color intensity (red, positive correlations; blue, negative correlation). ●statistically significant correlations (p-value < 0.05, up diagonal p-values adjusted with FDR correction).

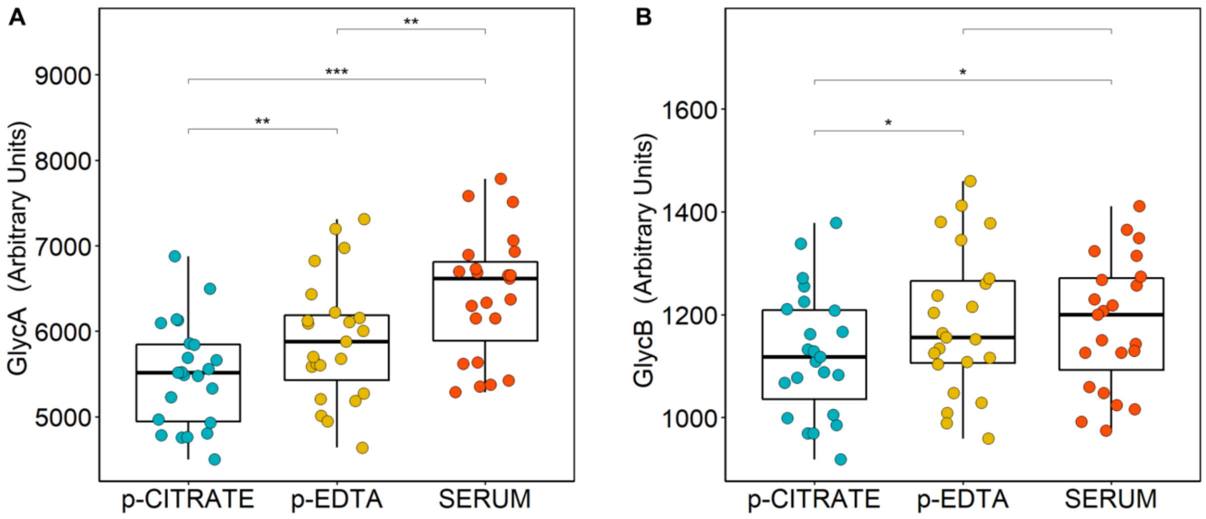

Figure S2. Boxplots of the integrated region of glycoproteins A) GlycA; B) GlycB. The different blood collection tubes are color-coded as follows: serum (red), EDTA plasma (yellow), and citrate plasma (blue). *P*-values adjusted for FDR are reported: *** $p < 0.001$; ** $p < 0.01$; * $p < 0.05$.
